# APOBEC3B Activity Is Prevalent in Urothelial Carcinoma Cells and Only Slightly Affected by LINE-1 Expression

**DOI:** 10.3389/fmicb.2018.02088

**Published:** 2018-09-04

**Authors:** Ananda Ayyappan Jaguva Vasudevan, Ulrike Kreimer, Wolfgang A. Schulz, Aikaterini Krikoni, Gerald G. Schumann, Dieter Häussinger, Carsten Münk, Wolfgang Goering

**Affiliations:** ^1^Department of Urology, Medical Faculty, Heinrich Heine University Düsseldorf, Düsseldorf, Germany; ^2^Clinic for Gastroenterology, Hepatology, and Infectiology, Medical Faculty, Heinrich Heine University Düsseldorf, Düsseldorf, Germany; ^3^Division of Medical Biotechnology, Paul-Ehrlich-Institut, Langen, Germany; ^4^Institute of Pathology, Medical Faculty, Heinrich Heine University Düsseldorf, Düsseldorf, Germany

**Keywords:** cytidine deaminase, APOBEC3B, APOBEC3G, APOBEC3H, urothelial cancer cells, LINE-1, innate immunity, mutation

## Abstract

The most common mutational signature in urothelial carcinoma (UC), the most common type of urinary bladder cancer is assumed to be caused by the misdirected activity of APOBEC3 (A3) cytidine deaminases, especially A3A or A3B, which are known to normally restrict the propagation of exogenous viruses and endogenous retroelements such as LINE-1 (L1). The involvement of A3 proteins in urothelial carcinogenesis is unexpected because, to date, UC is thought to be caused by chemical carcinogens rather than viral activity. Therefore, we explored the relationship between A3 expression and L1 activity, which is generally upregulated in UC. We found that UC cell lines highly express A3B and in some cases A3G, but not A3A, and exhibit corresponding cytidine deamination activity *in vitro*. While we observed evidence suggesting that L1 expression has a weak positive effect on A3B and A3G expression and A3B promoter activity, neither efficient siRNA-mediated knockdown nor overexpression of functional L1 elements affected catalytic activity of A3 proteins consistently. However, L1 knockdown diminished proliferation of a UC cell line exhibiting robust endogenous L1 expression, but had little impact on a cell line with low L1 expression levels. Our results indicate that UC cells express A3B at levels exceeding A3A levels by far, making A3B the prime candidate for causing genomic mutations. Our data provide evidence that L1 activation constitutes only a minor and negligible factor involved in induction or upregulation of endogenous A3 expression in UC.

## Introduction

The apolipoprotein B mRNA editing enzyme catalytic polypeptide 3 (APOBEC3, A3) protein family of Zn^2+^-dependent DNA cytidine deaminases constitutes a defensive network of proteins restricting exogenous viruses (Chiu and Greene, 2008; Harris and Dudley, 2015) and endogenous transposable elements (Schumann, 2007; Schumann et al., 2010; Refsland and Harris, 2013; Salter et al., 2016). They restrain retroviral replication mainly by deamination of cytidines in ssDNA following reverse transcription (Chiu and Greene, 2008; Münk et al., 2012; Vasudevan et al., 2013; Harris and Dudley, 2015). Importantly, APOBEC3B (A3B) is constitutively localized in the nucleus (Muckenfuss et al., 2006) and inhibits HIV-1 infection independent of the presence of Vif, which otherwise counteracts the activity of the remaining A3 family members (Bishop et al., 2004; Doehle et al., 2005). A3 proteins also inhibit human papilloma virus (HPV) and hepatitis B virus (HBV) (Harris and Dudley, 2015; Henderson and Fenton, 2015). Recently, large-scale exome and whole genome mutation studies have revealed distinct differences in mutational spectra and mutation frequencies between tumor entities (Alexandrov et al., 2013; Burns et al., 2013; Lawrence et al., 2013; Roberts et al., 2013). Many tumors of diverse entities display a characteristic mutational signature with strand-coordinated clusters of C→T transitions, which are frequently located in the proximity of chromosomal breakpoints. This signature is often associated with increased *A3A* or *A3B* mRNA expression levels and is thought to be caused by misdirected A3 activity, partly in conjunction with viral infection (Burns et al., 2013; Roberts et al., 2013). Indeed, almost all cervical cancers and a significant fraction of head and neck cancers (HNSCC), all harboring frequent A3-related mutations, are associated with viral infections (Vartanian et al., 2008; Lawrence et al., 2013). Additionally, A3G expression in HPV-induced uterine cervical intraepithelial neoplasia (CIN) and infiltration of A3G expressing CD3 positive T cells in CIN lesions were reported (Iizuka et al., 2017). In contrast, the frequent occurrence of a characteristic mutational A3 signature (Lawrence et al., 2013) in urothelial carcinoma (UC) is puzzling, as these tumors are thought to be caused predominantly by chemical carcinogens rather than viral infections (Tolstov et al., 2014).

It is assumed that retroelements, including endogenous retroviruses that are flanked by long terminal repeats (LTRs), and non-LTR retrotransposons such as long interspersed nuclear element-1 (LINE-1, L1) and short interspersed nuclear elements (SINEs), have been the original targets of A3 activity and have provided the evolutionary pressure necessary for the continuous expansion of the *A3* locus in primates (Münk et al., 2012). Mobilization of these retroelements is restricted by the different members of the A3 protein family to protect the genome from deleterious retrotransposition events (Muckenfuss et al., 2006; Schumann, 2007; Chiu and Greene, 2008; Goodier and Kazazian, 2008; Horn et al., 2013; Orecchini et al., 2018). For instance, the role of A3B in intracellular defense against transposable element activity was recently demonstrated by a twofold to fourfold increase in retrotransposition efficiency of an engineered human L1 reporter after shRNA-based knockdown of A3B in hESCs (Wissing et al., 2011). Importantly, L1 retrotransposition has been detected during development and progression of many human cancer entities (Lee et al., 2012; Doucet-O’Hare et al., 2015; Ewing et al., 2015) (for review: Carreira et al., 2014; Goodier, 2014; Burns, 2017). In UC, the most common histological subtype of urinary bladder cancer, L1-mediated retrotransposition frequency has not been established to date. However, L1Hs elements were reported to be particularly strongly hypomethylated in UCs (Nusgen et al., 2015), full-length L1 (FL-L1) transcript levels are increased (Kreimer et al., 2013) and L1 ORF1 protein (ORF1p) can be detected in UC tissues (Rodic et al., 2014; Whongsiri et al., 2018). Beyond L1-mediated retrotransposition, L1-encoded gene products may contribute to carcinogenesis by other mechanisms, including the regulation of RNA–DNA hybrids (Sciamanna et al., 2014; Schwertz et al., 2018). Moreover, experimental L1 downregulation in colon carcinoma cells led to reduced mRNA levels of the catalytic telomerase subunit *hTERT* and the telomerase RNA component *hTERC* (Aschacher et al., 2016). Whether this observation can be extrapolated to other cancer types like UC is so far unknown.

Conceivably, A3-induced genomic mutations may represent collateral damage to the human genome by a response originally directed against endogenous retrotransposons or exogenous viruses (Alexandrov et al., 2013). Thus, we hypothesized that in UC, where exogenous viruses are considered to contribute rarely to carcinogenesis, induction of A3 protein expression and their mutagenic effects might rather represent a response to the well-documented activation of endogenous L1 retrotransposons in these tumors. To address this hypothesis, we analyzed the mRNA expression profile of the different A3 family members in UC cell lines, which we had previously characterized for FL-L1 expression (Kreimer et al., 2013) and established the actual presence of A3-specific enzymatic activity. Subsequently, we investigated the consequences of modulating L1Hs expression by siRNA-mediated knockdown or ectopic overexpression for A3 expression and cellular properties. While UC cell lines did not express detectable *A3A* mRNA levels, expression of *A3B* mRNA was prominent in many. Our experiments provide evidence that there is only some minor effect of L1Hs expression on A3B promoter activity, which alone cannot explain the extensive upregulation of A3B expression in UC tissues and cell lines. Modulation of L1 expression did not have any consistently detectable effect on the expression of endogenous A3A, A3B, or A3G, even though knockdown of L1 elements with intact ORF1p impeded cell growth.

## Materials and Methods

### Tissue Samples and Cell Lines

All urothelial cancer cell lines (UCCs) used in this study (253J, 5637, 639-V, 647-V, BFTC905, HT-1376, J82, MGHU4, RT4, RT-112, SCaBER, SD, SW-1710, UMUC3, UMUC6, VM-CUB1, T24) were cultured in DMEM GlutaMax (Gibco, Darmstadt, Germany), supplemented with 10% fetal calf serum (Koch et al., 2012). BC61 cells were cultured as described previously (Seifert et al., 2007). The cell lines were obtained from the Leibniz Institute DSMZ-German Collection of Microorganisms and Cell Cultures (Braunschweig, Germany), except for the cell line UMUC3, which was kindly provided by Dr. Grossman (Houston, TX, United States). The human embryonal carcinoma cell lines NCCIT (ATCC CRL-2073) and Tera-1 (ATCC HTB-105) were kindly provided by Dr. R. Loewer, (Paul-Ehrlich-Institut, Langen, Germany) and cultured as described (Hoffmann et al., 2006). HeLa cells (ATCC CCL-2) were cultured following supplier’s recommendations. The telomerase-immortalized TERT-NHUC cell line was kindly provided by Dr. M. A. Knowles (Leeds, United Kingdom) and cultured as described (Chapman et al., 2009). Cell lines were authenticated prior to use by STR profiling in the Institute of Forensic Medicine, Heinrich Heine University Düsseldorf, Germany. Cultures of primary urothelial (UP) cells were established from ureters after nephrectomy and were routinely maintained in keratinocyte serum-free medium (KSFM, Gibco, Darmstadt, Germany) supplemented with 12.5 μg/ml bovine pituitary extract and 0.25 ng/ml epidermal growth factor as described (Swiatkowski et al., 2003). Tissue samples for UP generation were collected with patient informed consent and approval by the ethics committee of the medical faculty of the Heinrich Heine University, Study Number 1788.

### Nucleic Acid Extraction and cDNA Synthesis

To minimize DNA contamination, total RNA was extracted by acid phenol extraction followed by column purification. Synthesis of complementary DNA was performed using the QuantiTect Reverse Transcription Kit (Qiagen, Hilden, Germany), according to the manufacturer’s instructions, including an extra DNA removal step by DNase as recommended by the supplier. Briefly, 1 μg of total RNA was subjected to genomic DNA elimination reaction in a 14 μl volume, comprised of 2 μl of a 7x gDNA-Wipeout-Buffer, RNA, and water. The reaction mixture was incubated at 42°C for 2 min and then kept on ice. One microliter of the reaction mixture was taken and mixed with 14.38 μl of water in a new tube (considering 1 μg total RNA input, the RNA concentration in this solution would be 4.64 ng/μl), which served as mock RT template for RT-qPCR assay. With the remaining 13 μl reaction mixture, cDNA synthesis was performed (20 μl volume reaction mixture is made up of 1 μl RT, 4 μl RT buffer (5x), 1 μl RT primer mix, 1 μl water, and 13 μl DNAse treated RNA) by incubating the RT reaction components for 30 min at 42°C and then inactivating the RT enzyme by boiling for 95°C for 3 min (according to the manufacturer’s instruction). In order to minimize potential inhibitory effects of the RT buffer system on qPCR, a 1:10 dilution of the cDNA product was generated prior to the PCR reaction quantifying FL-L1 transcripts. The final nucleic acids concentration of the RNA suspension (used for the Mock RT-qPCR) and the cDNA suspension were both adjusted to 4.64 ng/μl prior to qPCR.

The efficiency of DNAse treatment was assessed by qPCR on RNA samples that were not incubated with any Reverse Transcriptase before. Data (Ct values) obtained from cell lines 5637, VM-CUB1, 639-V, SD, BC61, RT4 are provided in **Supplementary Table 1**. Ct values obtained from mock-RT experiments were found to be comparable with those obtained from blank control (water). The qPCR conditions were as follows: initial denaturation step at 95°C for 15 min, followed by 40 amplification cycles consisting of denaturation at 95°C for 15 s, annealing at 55°C for 20 s and extension at 72°C for 30 s, using the primers presented in the following method section and **Supplementary Table 2**.

### Quantitative Real-Time Reverse Transcription PCR (RT-qPCR)

RT-qPCR was performed on a 7500 Fast Real-Time PCR System (Applied Biosystems, Carlsbad, CA, United States) or Roche LightCycler 96 (Hoffmann-La Roche Ltd., Basel, Switzerland) using the QuantiTect SYBR Green PCR Kit (Qiagen, Hilden) with cDNA (1:10 diluted) from DNAse-treated RNA samples (see also above) as described previously (Goering et al., 2011). To quantify transcripts, specifically designed primers (**Supplementary Table 2**) were employed using the following PCR conditions: initial denaturation at 95°C for 15 min, followed by 40 amplification cycles consisting of denaturation at 95°C for 15 s, annealing at the appropriate temperature for 20 s and extension at 72°C for 30 s. Assay specificity was controlled for by using UCSC In-Silico PCR and melting curve profiles. All measurements were performed at least in duplicates; assay variance was <10%. Relative expression was calculated by the modified ΔΔCt method using TATA-box binding protein (TBP) mRNA levels as a reference gene transcript (Pfaffl, 2001). To ascertain efficient amplification, a standard curve was carried in each RT-qPCR experiment using cDNAs from activated PBMCs (*A3A*, *A3F*, *A3H*), UMUC3 (*A3B*, *A3D*), PC3 (*A3C*, *TBP*), 5637 (*A3G*), and VM-CUB1 (FL-L1), respectively.

To quantify transcript levels of human endogenous FL-L1 elements, primers specific for the 5′-UTR sequence of the L1.3 reference element (Acc. No. L19088.1, Sassaman et al., 1997) were used which bind L1.3 nucleotide positions 99–120 (L1_5′_for: 5′-GTACCGGGTTCATCTCACTAGG-3′) and 323–344 (L1-5′_rev: 5′-TGTGGGATATAGTCTCGTGGTG-3′) (**Supplementary Table 2**). RT-qPCRs with these primers were performed as previously described (Kreimer et al., 2013).

### Immunoblot Analysis

Twenty micrograms of each protein lysate were boiled in 3x SDS sample buffer (New England Biolabs, Frankfurt/Main, Germany), loaded on 4–12% Bis/Tris gels (Invitrogen), subjected to SDS-PAGE, and electroblotted onto nitrocellulose membranes. After protein transfer, membranes were blocked for 2 h at room temperature in 10% non-fat milk powder in 1xPBS-T [137 mM NaCl, 3 mM KCl, 16.5 mM Na_2_HPO_4_, 1.5 mM KH_2_PO_4_, 0.05% Tween 20 (Sigma-Aldrich, Mannheim, Germany)], washed in 1xPBS-T, and incubated overnight with the respective primary antibody at 4°C.

L1 ORF1p was detected using the polyclonal rabbit-anti-L1 ORF1p antibody #984 (Raiz et al., 2012) at a 1:2,000 dilution in 1xPBS-T containing 5% milk powder as primary antibody. Subsequently, membranes were washed three times in 1xPBS-T. As secondary antibodies, we used HRP-conjugated donkey anti-rabbit IgG antibody at a 1:30,000 dilution (Amersham Biosciences, Freiburg, Germany) in 1xPBS/5% milk powder for 2 h. Subsequently, the membrane was washed three times for 10 min in 1xPBS-T. β-actin expression was detected using a monoclonal anti-β-actin antibody (clone AC-74, Sigma-Aldrich, Steinheim, Germany) at a dilution of 1:30,000 as primary antibody and an anti-mouse HRP-linked species-specific antibody (from sheep) at a dilution of 1:10,000 as secondary antibody. Immunocomplexes were visualized using lumino-based ECL immunoblot reagent (Amersham Biosciences). For the immunoblot analysis shown in **Figure 2D**, the applied monoclonal anti-L1 ORF1p antibody was kindly provided by Dr. K. Burns (Johns Hopkins University, Baltimore, MD, United States) (Rodic et al., 2014) or purchased (clone 4H1, 1:10,000 dilution, Millipore, Darmstadt, Germany). For immunoblot detection of A3H protein in selected UCCs, anti-Human APOBEC3H monoclonal antibody (P1H6, cat # 12156, 1:10^3^ dilution, NIH AIDS reagent) was used.

### Transfection Experiments

In order to knockdown expression of functional endogenous L1 elements, cells were transfected for 72 h with 20 nmol of either L1_siRNA#1 (5′-GAGAACGCCACAAAGAUACtt-3′) (Oricchio et al., 2007) or L1_siRNA#2 (5′-GAAAUGAAGCGAGAAGGGAAGUUUA-3′) (Aschacher et al., 2016) targeting specifically nucleotide positions 1512–1531 or 1287–1312 of the L1.3 reference sequence (acc.no: L19088.1; Sassaman et al., 1997), respectively, (**Supplementary Table 2**) or a non-specific control (Silencer select negative control siRNA 1; Life Technologies, Darmstadt, Germany). Transfections were performed using Lipofectamine RNAiMAX (Life Technologies) according to the manufacturer’s instructions. The chosen siRNAs specifically target roughly 500 full-length and truncated L1 elements in the average human genome of L1PA1 subfamily L1Hs sequences, which can be transcriptionally active (Skowronski et al., 1988; Sheen et al., 2000; Tang et al., 2017). To ensure that the siRNA effects persist in long-term (120 h) experiments, the siRNA transfection procedure was repeated 3 days after the initial transfection. Knockdown of *A3B* and *A3G* expression was accomplished by transient transfection of siRNAs (Cat. Nos. L-017322-00 and L-013072-00, Dharmacon/ON-TARGETplus siRNA Reagents) for 72 h. As indicated in **Figure 4**, the total concentration of siRNAs was maintained at 20 nM for all transfections.

The episomal L1 retrotransposition reporter plasmids pJM101/L1_RP_ (Kimberland et al., 1999) and pAJG101/L1_RP_ (**Supplementary Figure 1**) facilitating ectopic expression of the *mneo*I-tagged, full-length L1_RP_ element, and their empty vector pCEP4 (Thermo Fisher Scientific) were separately transfected into UC cells using the X-tremeGENE 9 DNA transfection reagent (Roche). In pAJG101/L1_RP_ the CMV promoter in pJM101/L1_RP_ was replaced by the CAG promoter (Niwa et al., 1991; Kobayashi, 1996).

### A3B Promoter Constructs and Reporter Assays

For this study, we have constructed two *A3B* promoter luciferase reporter constructs pA3B-120 and pA3B-1200 (**Supplementary Figure 2**). To generate pA3B-1200, the genomic sequence flanked by nucleotide position -1200 and +18 relative to the ATG start codon of the *A3B* gene was amplified from genomic HeLa DNA applying forward primer A3B_Pr_-1200_F (5′-GATGGTACCGCTCCCAGCAACCCCCCAG) and reverse primer A3B_Pr_-1200_R (5′-CATGCTAGCCTGATCTGTGGATTCATGTTCAGC) and Pwo DNA polymerase (Roche) using the following PCR conditions: one cycle 94°C for 2 min; 30 cycles of 94°C for 30 s, 58°C for 60 s, and 72°C for 60 s; one cycle 72°C for 7 min. The amplicon was inserted into the promoterless luciferase reporter plasmid pGL3-Basic (Promega, Mannheim, Germany) via KpnI and NheI restriction sites in the primer sequences (**Supplementary Figure 2**).

pGL3-Basic (Promega) derived reporter plasmid pA3B-120 containing a 120-bp fragments of the A3B promoter was constructed from pA3B-1200 using forward primer A3B_Pr_-120_F (5′-GATGGTACCGCCCTGGGAGGTCACTTTAAG) and reverse primer A3B_Pr_-1200_R (**Supplementary Figure 2**). UC cells 5637 and VM-CUB1 were plated in 24-well dishes and cotransfected with either pA3B-120 or pA3B-1200 and either pJM101/L1_RP_ or pAJG101/L1_RP_ on the next day using X-tremeGENE 9 DNA transfection reagent (Roche). The cells were cotransfected with each A3B-promoter-luciferase reporter construct or the ΔHOX plasmid, containing a frameshifted HOXB13 cDNA in pcDNA/TO4, as a MOCK-transfection control. Luciferase activity was quantified 2 days post transfection using the Dual Luciferase Reporter Assay System (Promega, Mannheim, Germany) as described (Goering et al., 2011).

### APOBEC3 Detection and *in vitro* DNA Deamination Assay

HEK293T cells were transfected with plasmids expressing hemagglutinin (HA)-tagged A3B or A3G using Lipofectamine LTX (Thermo Fisher Scientific). 5637, UMUC3 and VM-CUB1 UCCs were transfected with the pJM101/L1_RP_ expression construct or ΔHOX as a MOCK-transfection control as described above. Seventy-two hours later, cells were washed in PBS and lysed with a mild lysis buffer [50 mM Tris (pH 8), 1 mM PMSF, 10% glycerol, 0.8% NP-40, 150 mM NaCl and 1X complete protease inhibitor (Calbiochem, Darmstadt, Germany)]. Lysates were clarified by centrifugation for 20 min at 14,800 rpm at 4°C. For immunoblot analyses, samples were boiled at 95°C for 5 min in Roti load reducing buffer (Roth, Karlsruhe, Germany), separated on an SDS-PAGE gel followed by transfer to a PVDF membrane. Membranes were blocked in TBST containing 5% skimmed milk powder and probed with the respective primary antibody. A rabbit anti-A3G antibody (anti-ApoC17; 1:10^4^ dilution, NIH AIDS reagent) that is cross-reactive against A3A and A3B was used to detect A3B and A3G proteins (Mitra et al., 2014; Jaguva Vasudevan et al., 2018). Mouse anti-α-tubulin antibody (1:4,000 dilution, clone B5-1-2; Sigma-Aldrich, Taufkirchen, Germany), or goat anti-GAPDH (C-terminus, 1:15,000 dilution, Everest Biotech, Oxfordshire, United Kingdom) were used as primary antibodies for loading controls.

*In vitro* deamination reactions were performed as described (Nowarski et al., 2008; Jaguva Vasudevan et al., 2013) in a 10 μl reaction volume containing 25 mM Tris pH 7.0, and 100 fmol single-stranded DNA substrate (CCCA : 5′-GGATTGGTTGGTTATTTGTTTAAGGAAGGTGGATTAAAGGCCCAAGAAGGTGATGGAAGTTATGTTTGGTAGATTGATGG; TTCA: 5′-GGATTGGTTGGTTATTTGTATAAGGAAGGTGGATTGAAGGTTCAAGAAGGTGATGGAAGTTATGTTTGGTAGATTGATGG) with 2 μl of freshly prepared cell lysate. Samples were divided in half and 50 μg/ml RNase A (Thermo Fisher Scientific) were added to one half. Subsequently, reactions were incubated for 1 h at 37°C and the reaction was terminated by incubating samples at 95°C for 5 min. An equivalent of one fmol single-stranded DNA substrate was used for PCR amplification [Dream Taq polymerase (Thermo Scientific)] comprising 95°C for 3 min, followed by 30 cycles of 61°C for 30 s and 94°C for 30 s) using the forward primers 5′-GATTGGTTGGTTATTTGTTTAAGGA for the CCCA substrate or 5′-GGATTGGTTGGTTATTTGTATAAGGA for the TTCA substrate, and in both cases the reverse primer 5′-CCATCAATCTACCAAACATAACTTCCA. PCR products resulting from the CCCA and TTCA substrates were digested with the restriction enzymes Eco147I (StuI) (Thermo Fisher Scientific) or MseI (NEB, Frankfurt/Main, Germany), respectively, and the resulting restriction fragments were separated on a 15% native PAGE gel and stained with ethidium bromide solution (7.5 μg/ml). To control for successful and efficient restriction digestion of the PCR products, additional substrate oligonucleotides in which the nucleotide sequences 5′-CCCA-3′ and 5′-TTCA-3′ were replaced by 5′-CCUA-3′ and 5′-TTUA-3′, respectively, were digested in parallel.

### Cell Viability, Proliferation, Apoptosis, and Senescence-Associated (SA)-β-Galactosidase Assays

Cell viability and apoptosis were assessed in quadruplicates by CellTiter-Glo^®^ Luminescent Cell Viability Assay and Caspase-Glo^®^ 3/7 Assay (Promega, Madison, WI, United States), respectively, using a Wallac 1420 VICTOR2^TM^ plate reader (PerkinElmer, Waltham, MA, United States). Cell proliferation was measured by an EdU incorporation assay (baseclick GmbH, Neuried, Germany), according to the manufacturer’s instructions. The SA-β-galactosidase assay was performed as described (Itahana et al., 2007).

### Clonogenicity Assay

For clonogenicity assays, cells were plated at low density into 6 cm dishes and grown until colonies started to become confluent for the fastest growth condition. Colonies were stained with Giemsa (Merck, Darmstadt, Germany).

### Analysis of *A3H* Single Nucleotide Polymorphism (SNP)

Since *A3H* haplotype I encodes an active cytosine deaminase described to reside in the nucleus and linked to cancer mutagenesis (Starrett et al., 2016), the status of *A3H* SNPs was determined in our panel of UCCs. A 305-bp genomic DNA genomic DNA fragment of the *A3H* gene was amplified from DNAs from various UCCs using primer pairs, A3H forward 5′-AGTGCCATGCAGAAATTTGCTTT and A3H reverse 5′-CGGGGGTTTGCACTCTTATAACT. Amplified fragments were subjected to direct Sanger sequencing and results were analyzed for the highly polymorphic SNP rs139297 and the adjacent SNPs rs139298/rs139299.

### Statistical Analyses

*p*-Values were calculated by the Mann–Whitney *U* Test using SPSS Statistics 21 (IBM, Armonk, NY, United States) the unpaired Student’s *t*-test with Graphpad Prism (GraphPad Software, San Diego, CA, United States). Data were represented as the mean ± standard deviation (SD). Significant differences (*p* < 0.05) are marked by asterisks. Correlation coefficients and significance were calculated by non-parametric Spearman’s rank correlation (Spearman’s rho) and were corrected for multiple testing using the Bonferroni method.

## Results

### *A3B* Is Upregulated in Urothelial Cancer Cells

**FIGURE 1:**
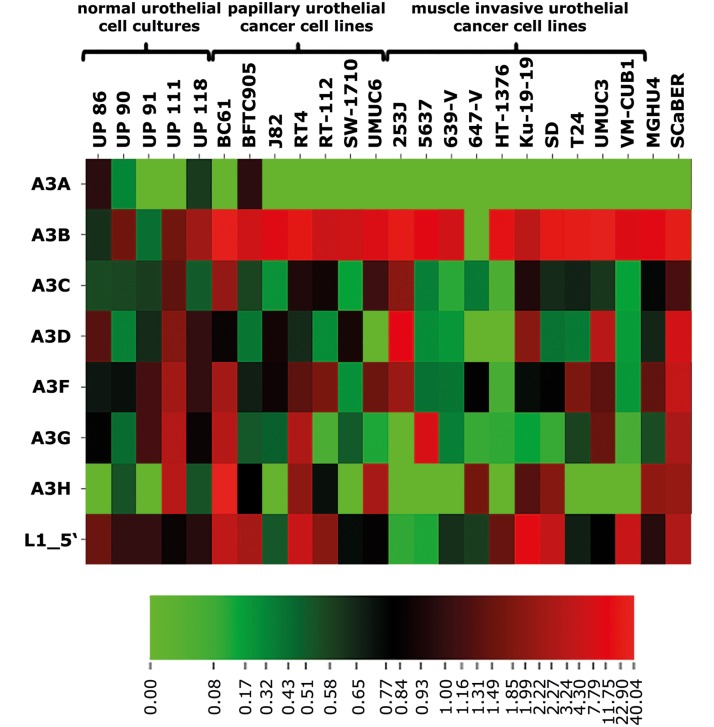
Heatmap representation of relative transcript levels of the seven members of the APOBEC3 family of cytidine deaminases and of endogenous FL-L1 elements. Expression of *A3A* to *A3H* was quantified by RT-qPCR in five individual urothelial cell cultures (UP86 to UP118), 17 papillary and muscle-invasive urothelial cancer cell (UCC) lines (BC61 to VM-CUB1) and two squamous urothelial cancer cell lines (MGHU4; SCaBER). Relative expression of each *A3* gene and of L1 transcript levels was calculated using the TATA-box binding protein (TBP) mRNA levels as a reference transcript and median expression of UPs were set as 1. L1 transcript levels were obtained from Kreimer et al. (2013). For the heatmap representation, relative expression values ranging from 0 to 40.04 were binned for each row and transformed in color-code indicating low (green) to high (red) expression. For a bar chart representation of relative A3 transcript levels presented in this Figure, see **Supplementary Figure 3**.

As a first step to investigate if the expression of specific members of the A3 protein family of cytidine deaminases is a response to the upregulation of endogenous L1 expression, we profiled mRNA levels of all seven members of the A3 protein family (*A3A*, *A3B*, *A3C*, *A3D*, *A3F*, *A3G*, and *A3H*) in 19 UCCs and five independent primary cultures of normal urothelial cells (UPs) by RT-qPCR. *A3A* was only detectable in the urothelial cell culture UP86, the UCC BFTC905 (**Figure 1** and **Supplementary Figure 3**) and in activated PBMCs serving as positive control for *A3A* expression (data not shown). *A3A* expression was not detectable in all remaining UCCs and UPs. In contrast, *A3B* expression was barely detectable in all analyzed urothelial cell cultures, but was high in all analyzed UCCs except for the 647-V line harboring no detectable *A3B* mRNA at all (**Figure 1** and **Supplementary Figure 3**). *A3C*, *D*, and *F* transcripts were detectable at moderate levels in all tested UPs and the majority of UCCs, but a few UCCs expressed *A3C* and *A3D* at extremely low or undetectable levels (**Figure 1** and **Supplementary Figure 3**). *A3G* expression was robust in UPs but low in most UCCs, especially in those originating from muscle-invasive urothelial cancers (**Figure 1** and **Supplementary Figure 3**). *A3H* transcripts were below detection level in the majority of muscle-invasive UCCs and in two UPs, while the remaining cell lines and UP cultures displayed moderate or robust transcript levels. In the papillary UCC BC61, *A3H* expression was exceptionally upregulated (**Figure 1** and **Supplementary Figure 3**).

Quantification of FL-L1 transcripts revealed moderate L1 expression in all UPs and robust transcriptional L1 upregulation in almost all analyzed UCCs with the exception of the UCCs 253J and 5637 (**Figure 1** and **Supplementary Figure 3**). If L1 upregulation contributed to activation of A3 enzymes, mRNA expression of L1 and *A3* would be expected to correlate. We therefore calculated whether any correlation between FL-L1 mRNA expression levels and mRNA levels of the various *A3* genes could be detected in UCCs using Spearmen’s rho correlation (**Figure 1**, see also Kreimer et al., 2013). Surprisingly, only the correlation between L1 and *A3H* RNA levels reached the level of significance (Spearman’s rho 0.419, ^∗^*p* = 0.042), which was lost after Bonferroni correction for multiple testing.

Because *A3H* was expressed in several UPs and *A3H* haplotype I (*A3H-I*), a specific allele of *A3H*, has been implicated in breast and lung carcinogenesis (Starrett et al., 2016), we additionally determined the *A3H* genotype at SNPs rs139297, rs139298 and rs139299 in UCCs (**Supplementary Table 3**). Approximately two thirds of the tested UCCs harbor the G105/K121 allele associated with the *A3H-I* haplotype in a homozygous (6/18) or heterozygous (7/18) manner. However, expression of A3H was not detectable in selected UCCs irrespective of the *A3H* genotype (**Supplementary Figure 7**). This finding implies *A3H* as a possible but unlikely source of A3 mutations in several but not all UCCs.

### L1 ORF1p Is Expressed in Most UCCs

The design of the L1-specific RT-qPCR assay to quantify L1 transcript levels (Kreimer et al., 2013; **Figure 1**) which is based on the L1.3 reference sequence (Sassaman et al., 1997), does not allow to fully distinguish transcripts of the approximately 100 retrotransposition-competent L1Hs elements (Brouha et al., 2003) encoding functional L1 ORF1 and L1 ORF2 proteins from non-functional FL-L1 transcripts. Therefore, to investigate relative expression levels of L1Hs elements encoding functional L1 proteins, we performed quantitative immunoblot analyses using anti-L1-ORF1p antibodies (Raiz et al., 2012; Rodic et al., 2014).

**FIGURE 2:**
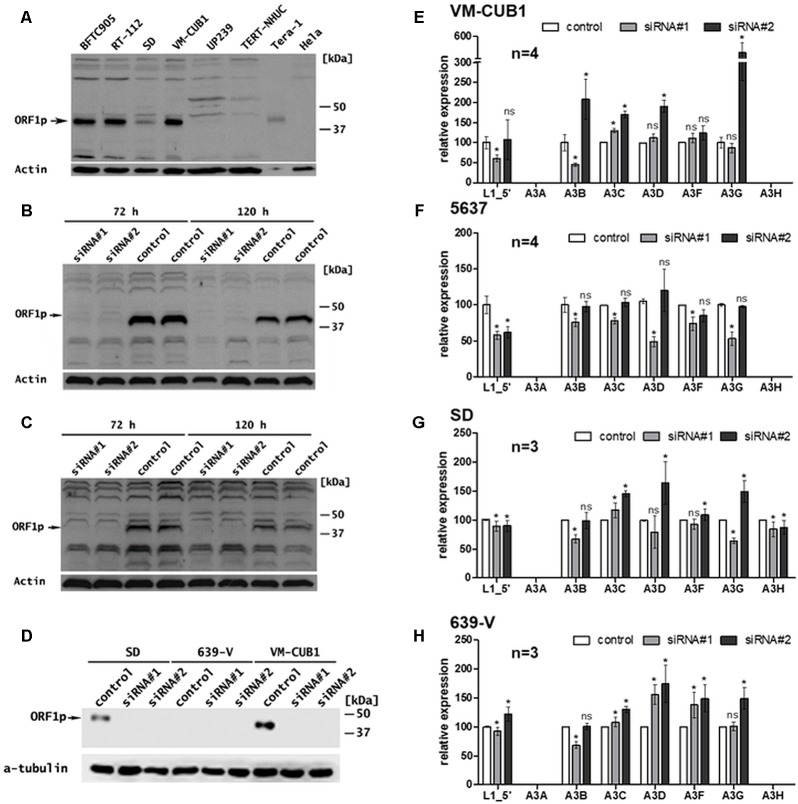
Effects of transcriptional knockdown of endogenous FL-L1 expression on transcription of the APOBEC3 gene family members in UCCs. **(A)** L1 ORF1p expression was analyzed in selected UCCs (BFTC905, RT-112, SD, and VM-CUB1) and benign urothelial samples (UP239, TERT-NHUC) by immunoblot analyses using an anti-ORF1p antibody. Tera-1 (highly diluted) and HeLa protein extracts were used as positive and negative control for L1 ORF1p expression, respectively. L1 ORF1p expression was determined in VM-CUB1 **(B)**, 5637 **(C)**, SD, and 639-V **(D)** UCCs after treatment with 20 nM control or L1-specific siRNAs for 72 h **(B,C)** and 120 h **(B–D)** by immunoblot analysis. β-actin or tubulin protein was detected as loading control. Note longer exposure in **(C)**. In **(D)** VM-CUB1 served as a positive control for siRNA treatment. mRNA expression of L1, *A3A*, *A3B*, *A3C*, *A3D*, *A3F*, and *A3G* was assessed in VM-CUB1 **(E)**, 5637 **(F)**, SD **(G)**, and 639-V **(H)** UCCs by RT-qPCR after 72 h treatment with 20 nM LINE-1 specific siRNAs and control siRNA. Relative expression levels were calculated using TBP mRNA levels as a reference transcript and expression in control siRNA treated samples were set as 100. “n” represents the number of independent knock down experiments. Data were represented as means ± standard deviations (error bars). *P*-values were calculated using *t*-tests. Asterisk represents statistically significant difference: ^∗^*P* < 0.05 and ns, not significant.

Consistent with their previously assessed relative FL-L1 mRNA levels (Kreimer et al., 2013) (**Figure 1**), elevated amounts of L1 ORF1p were detected in the UCC lines BFTC905, RT-112, VM-CUB1, and SD (**Figure 2A** and **Supplementary Figures 4A,C**). More moderate but still detectable L1 ORF1p levels were present in J82, SW-I710, UMUC6, 253J, 5637, 639-V, 647-V, HT-1376, T24, and UMUC3 cells (**Supplementary Figure 4**). The relatively modest amounts of L1 ORF1p in BC61 and RT4 cells do not seem consistent with the transcriptional L1 upregulation in these cells (**Figure 1**), but could be explained by the fact that a subset of FL-L1 elements transcribed in these cell lines does not encode intact L1 proteins. Of note, L1 ORF1p expression was not detectable in the normal urothelial cell culture UP239 and the TERT-NHUC immortalized normal urothelial cell line (**Figure 2A**).

### L1 siRNA-Mediated Knock Down of Functional, Endogenous L1 Elements in UCCs Exert Largely Diverse Effects on A3 Expression

In order to investigate potential effects of L1-encoded gene products on the expression of A3 proteins, we modulated the expression of L1 elements in selected representative UCCs with robust endogenous A3B transcription levels and either low/moderate (5637 and 639-V) or high (VM-CUB1 and SD) L1 mRNA and ORF1p expression levels, respectively. Expression of full-length L1Hs elements was downregulated in the four UCCs by transfecting either siRNA#1 (Oricchio et al., 2007) or siRNA#2 (Aschacher et al., 2016), which are specifically directed against ORF1 of the human L1.3 reference sequence (Sassaman et al., 1997; see Materials and Methods).

Transfection of each of the two L1-specific siRNAs efficiently knocked down L1 ORF1p expression in VM-CUB1 (**Figures 2B,D**), 5637 (**Figure 2C**), and SD UCCs. L1 mRNA and ORF1p expression was barely detectable in 639-V and remained unchanged after L1 siRNA treatment (**Figures 1**, **2D**). Interestingly, RT-qPCR using primer combinations specific for the 5′end of the L1 5′-UTR (see Materials and Methods and **Supplementary Table 2**) revealed that overall FL-L1 transcript levels were reduced only by at most 50% (**Figures 2E–H**). Since the partial mRNA knockdown observed in these cell lines nevertheless resulted in a highly efficient L1 ORF1p depletion (**Figures 2A–D**), this observation indicates that the siRNAs target most if not all intact protein-coding L1 elements harboring an intact ORF1 efficiently. In contrast, non-functional FL-L1 elements with mutations in ORF1 that differ from the L1.3 reference sequence were targeted less efficiently.

Following transfection with L1-specific siRNAs, *A3A* mRNA expression remained undetectable in all four UCC lines (**Figures 2E–H**). *A3H* expression was only detectable in SD cells among these four UCCs (compare with **Figure 1**) and not altered by the knockdown of L1 expression in the examined UCCs (**Figures 2E–H**), whereas L1 knockdown exerted variable effects on the expression of *A3B* to *A3G* in the tested UCCs.

Transfection of L1_siRNA#1 diminished overall FL-L1 transcript levels in the analyzed UCC lines by 8% (639-V) to 42% (5637) (**Figures 2E–H**), and was associated with a drop of *A3B* expression by 25% (5637) to 55% (VM-CUB1). *A3G* expression was diminished by 14, 47 and 36% in VM-CUB1, 5637, and SD cells, respectively, and remained unaffected in 639-V cells. However, only minor, negligible effects on *A3C*, *A3D* and *A3F* transcript levels were observed in VM-CUB1, 5637, and SD cells. In 639-V cells, *A3C* expression was unchanged, whereas *A3D* and *A3F* transcript levels were increased by 56% and 38%, respectively.

Following transfection with L1_siRNA#2, *A3B* transcript levels remained basically unchanged in 5637, SD, and 639-V cells, but increased in VM-CUB1 cells by approximately twofold. *A3C* expression was increased in VM-CUB1, SD, and 639-V cells by 70%, 45%, and 30%, respectively, but remained unchanged in 5637 cells. An increase in *A3D* expression was observed in each of the four UCCs and ranged from 12 to 90%, while *A3F* transcript levels were upregulated only in VM-CUB1 and 639-V cells, and remained essentially unaffected in 5637 and SD cells (**Figures 2E–H**). The knockdown of functional L1 elements was associated with an increase of *A3F* transcript levels in 639-V cells by 44%, but had no meaningful effect on *A3F* expression in the remaining cell lines. L1_siRNA#2-mediated knockdown of ORF1p-expressing endogenous L1 elements was associated with a strong increase of *A3G* transcript levels in three UCCs ranging from 50% in the SD line to fourfold in VM-CUB1 cells but had no consequences for *A3F* expression in 5637 cells, and no noteworthy effect on 5637 cells (**Figures 2E–H**).

In summary, knockdown of functional L1 elements with L1_siRNA#2 resulted in a general increase of *A3C*, *D*, *F*, and *G* transcript levels in three out of four analyzed UCCs, while L1_siRNA#1-mediated knockdown was associated with a comparable major increase in *A3C*, *A3D*, and *A3F* transcript levels only in 639-V cells and a minor increase in VM-CUB1 cells. A decrease of *A3* transcript levels was observed only after L1_siRNA#1-mediated knockdown in 5637 and SD UCCs and was minor, ranging from 10 to 50% among the *A3* genes. Taken together, no consistent correlation between the knockdown of functional endogenous L1 elements and expression of *A3* genes could be observed, but knockdown of intact L1s was more often associated with increases rather than decreases of *A3* gene expression. In sum, these findings are not consistent with the hypothesis that expression of *A3* genes is upregulated in UCCs as a consequence of functional L1s activation. Furthermore, it is currently unclear why the effects of functional L1 knockdown on *A3* gene expression differed between L1_siRNA#1- and L1_siRNA#2-mediated knockdowns.

### Ectopic Expression of Retrotransposition-Competent L1 Elements Has Only Minor Effects on Expression of Selected A3s

**FIGURE 3:**
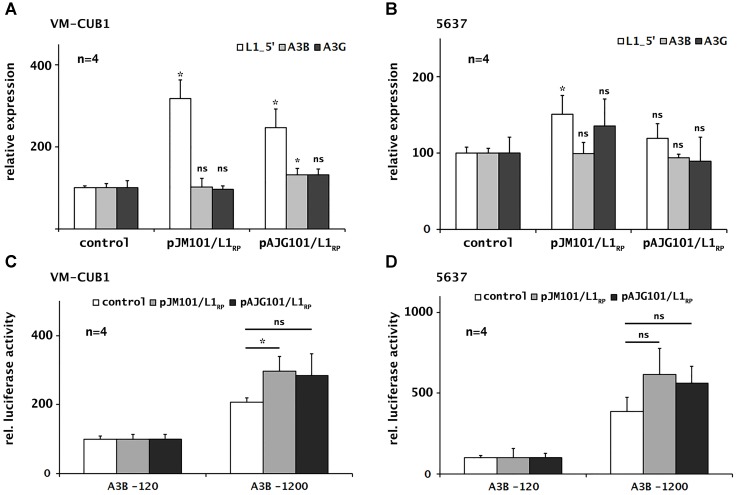
Consequences of ectopic L1 overexpression on endogenous *A3B* and *A3G* transcription and *A3B* promoter activity in selected UCCs. Expression of endogenous and transfected L1 elements, *A3B* and *A3G* was determined in UCCs VM-CUB1 **(A)** and 5637 **(B)** after transfection with L1 expression plasmids (pJM101/L1_RP_ or pAJG101/L1_RP_) or control plasmids using RT-qPCR. *A3B* promoter activity was assessed in VM-CUB1 **(C)** and 5637 **(D)** UCCs using luciferase reporter constructs coding for the *A3B* minimal promoter (A3B-120) or *A3B* full promoter (A3B-1200). Promoter constructs were co-transfected with pJM101/L1_RP_ or pAJG101/L1_RP_ expression plasmids or a control plasmid and luciferase activity was assessed subsequently. Luciferase activity values were normalized to luciferase activity data obtained from co-transfection of the A3B-120 minimal promoter construct with the L1 expression construct or the control plasmid, respectively, which were each set as 100. “n” represents the number of independent knock down experiments. Data were presented as means ± standard deviations (error bars). *P*-values were calculated using Mann–Whitney *U* Test. Asterisk represents statistically significant difference: ^∗^*P* < 0.05 and ns, not significant.

To investigate the consequences of ectopic overexpression of retrotransposition-competent L1 elements on endogenous *A3B* and *A3G* transcript levels in UCC lines, we next transfected either of the L1 expression plasmids pJM101/L1_RP_ and pAJG101/L1_RP_ (**Supplementary Figure 1**) into the cell lines VM-CUB1 and 5637, which are characterized by relatively high and low endogenous L1 transcript levels, respectively (**Figure 1**). Following transfection, FL-L1 RNA levels increased 2.5- to 3-fold in VM-CUB1 cells and by 23% to 46% in 5637 cells, as demonstrated by the L1_5′-UTR-specific RT-qPCR assay (**Figures 3A,B**). To analyze whether ectopic L1 overexpression affects endogenous *A3* expression, we quantified *A3A*, *A3B*, and *A3G* mRNA levels in the transfected UCCs. We found that expression of *A3B* and *A3G* was slightly increased in VM-CUB1 UCC after transfection with pAJG101/L1_RP_, but only *A3B* expression changes reached the level of significance (**Figures 3A,B**). In 5637 UCC, *A3B* and *A3G* expression was not altered significantly. Of note, *A3A* expression remained undetectable in both cell lines (data not shown).

To study whether ectopic expression of functional L1 elements can induce *A3B* promoter activity, we co-transfected VM-CUB1 and 5637 cells with either of the two A3B promoter luciferase reporter constructs pA3B-120 or pA3B-1200 (**Supplementary Figure 2**) together with the L1 expression plasmids pJM101/L1_RP_ and pAJG101/L1_RP_ or the empty pCEP4 vector as negative control. Activity of the *A3B* promoter encoded by pA3B-1200 increased by ∼36% – 42% and 64% – 80%, respectively, after co-transfection of the luciferase reporter construct with pJM101/L1_RP_ or pAJG101/L1_RP_ into VM-CUB1 and 5637 cells relative to the effect of the control vector (**Figures 3C,D**). This increase in promoter activity is consistent with the increase in endogenous *A3B* mRNA levels by ∼27% after transfection of plasmid pAJG101/L1_RP_ in VM-CUB1 cells (**Figure 3A**). Taken together, induction of L1 activity has only minor effects on A3B expression as well as promoter activity and significant effects are limited to VM-CUB1 UCC with high L1 expression.

### A3B Deaminase Activity Is Predominant in UCCs and Not Altered by Ectopic L1 Expression

**FIGURE 4:**
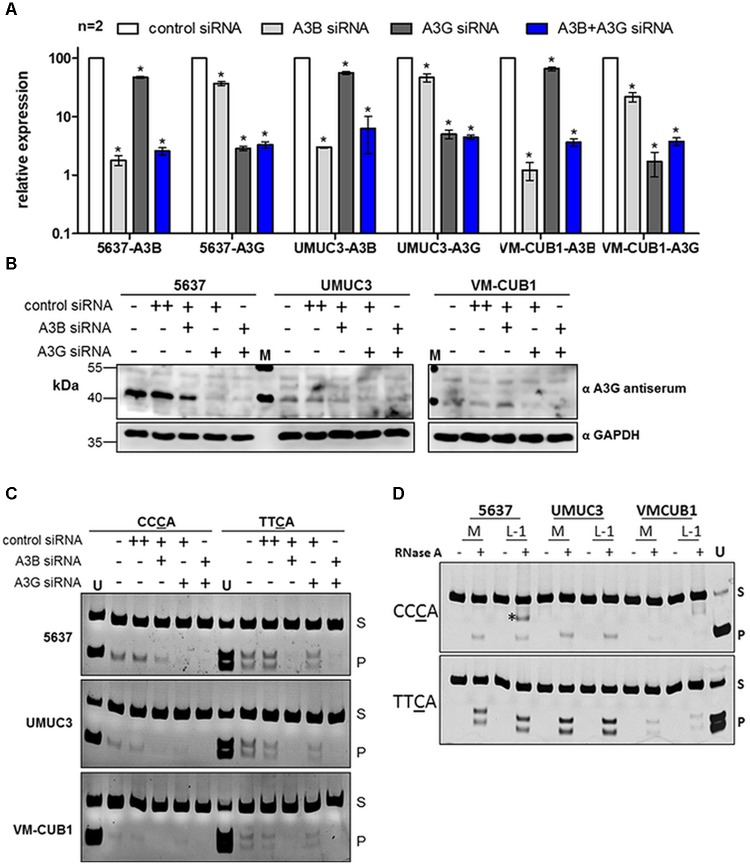
Expression and *in vitro* deamination activity of endogenous A3B and A3G in cell lysates of UC cell lines. **(A)** RT-qPCR analysis of *A3B* and *A3G* transcripts in 5637, UMUC3, and VM-CUB1 UCCs transfected with control, *A3B*- or *A3G*-specific siRNAs as indicated. UCCS were incubated for 72 h with a final siRNA concentration of 20 nM. Values are means ± standard deviations (error bars) obtained from two independent transfection experiments (*n* = 2). *P*-values were calculated using unpaired *t*-tests and statistical significant changes (*p* < 0.05) are indicated by asterisks (^∗^). **(B)** Immunoblot analysis of endogenous A3B and A3G expression in 5637, UMUC3, and VM-CUB1 UCCs after transfection with control, *A3B*- or *A3G*-specific siRNAs for 72 h as indicated. GAPDH expression served as loading control. M, molecular weight standard. **(C)** Deamination activity of siRNA-treated and untreated samples as described in **(B)**, was assessed by *in vitro* DNA deamination assay. The enzymatic activity of endogenous A3B and A3G proteins was tested on two different oligonucleotide substrates containing either the CCCA or TTCA motif. All reactions were treated with RNAse A to derive physiologically active A3 proteins from higher mass RNA complexes. Deamination product band (P) and substrate band (S) are marked. As a deamination product marker and as a restriction enzyme control, substrates containing the CCUA or TTUA motif were cleaved by their respective restriction enzyme and loaded on the gel (U). **(D)** Deamination activity of protein extracts isolated from UCCs 5637, UMUC3, and VM-CUB1 that were transfected with the Mock-control (M) or pAJG101/L1_RP_ plasmid was investigated. RNAse A-untreated and treated samples were included. “^∗^” indicates an unspecific band. To validate substrate-specific A3 deamination activity, the assay was performed using protein extracts from 293T cells previously transfected with A3B or A3G expression plasmids, respectively (**Supplementary Figure 5**).

We investigated expression and deaminase activity of A3 enzymes suspected to cause mutations during bladder carcinogenesis in selected UCCs with different *A3* mRNA expression levels. We chose specifically (i) the UCC 5637 exhibiting major transcriptional upregulation of *A3B* and *A3G*, (ii) UMUC3 showing robust transcript levels of most *A3* genes (with *A3B* expression being the highest), and (iii) VM-CUB1 with very low transcript levels of most *A3* family members except for *A3B* (**Figure 1**). Distinguishing A3B from A3G by standard immunoblotting techniques is challenging due to the high amino-acid sequence homology between both proteins and their comparable molecular masses (Burns et al., 2015; Jaguva Vasudevan et al., 2018). Therefore, to determine whether A3B or A3G is responsible for any potential deamination activity in these cancer cell lines, A3B and A3G were knocked down separately in different cultures or simultaneously in the same culture. Successful downregulation of *A3B* and *A3G* was confirmed by RT-qPCR using A3B- and A3G-specific primer pairs. Transfection of the UCCs 5637, UMUC3, and VM-CUB1 with *A3B*-specific siRNA alone reduced *A3B* expression by >90% (**Figure 4A**). Similarly, *A3G* expression levels dropped after transfection with *A3G*-specific siRNA to below 10% in all three UCCs (**Figure 4A**). Simultaneous treatment with *A3B*- and *A3G*-specific siRNAs resulted in diminished *A3B* and *A3G* mRNA levels in all examined UCCs comparable to those observed after single siRNA treatment (**Figure 4A**).

Immunoblot analyses of cell extracts isolated from the differently transfected 5637 cells with an anti-A3G antibody (ApoC17) (Kao et al., 2003) reported to cross-react with A3B, detected a ∼45 kDa protein in 5637 cells, which is consistent with the predicted molecular weights of both A3B and A3G proteins (**Figure 4B**) and their mRNA expression pattern in 5637 cells (**Figure 1**). The intensity of the 45 kDa band was slightly diminished after transfection of the cells with *A3B*-specific siRNA, but the band disappeared almost entirely after transfection with A3G-specific siRNA or a combination of both siRNAs (**Figure 4B**). Expression of the 45-kDa protein was not affected by transfection of control siRNA.

Transfection of *A3G*-specific siRNA strongly depleted the amounts of the 45-kDa protein in both UMUC3 and VM-CUB1 cells, whereas the A3B-specific siRNA had only a minor effect on the 45-kDa protein levels in UMUC3 cells and did not affect its expression at all in VM-CUB1 cells (**Figure 4B**). These findings suggest that the majority of the 45-kDa proteins detected with the anti-A3G antibody represents A3G. A note of caution on the detection of endogenous A3B: Since a more specific antibody capable of selectively detecting endogenous A3B enzyme in UC cell lines is currently not available, we cannot formally exclude that A3B protein is not depleted by *A3B*-specific siRNA, despite downregulation of *A3B* mRNA. Of note, A3A expression was not considered because there was no evidence for the presence of *A3A* mRNA in the analyzed UCC lines (**Figures 1**, **2**), and consistently, immunoblot analysis with anti-A3A antibodies did not provide any evidence for the presence of A3A proteins (data not shown).

In order to investigate if A3B and/or A3G are enzymatically active in UCC lines, DNA deamination activity assays were performed using cells lysates from the different UCCs transfected with *A3B*- and *A3G*-siRNAs as controls (**Figure 4C**). To measure deaminase activity, we applied a qualitative PCR-based *in vitro* DNA deamination assay to identify C→U conversion in an 80-nt single-stranded DNA substrate harboring the isozyme-specific motif TTCA or CCCA, specifically recognized by A3B or A3G, respectively (Jaguva Vasudevan et al., 2017; Yang et al., 2017). Catalytic deamination of C→U in the respective motif creates specific restriction sites, which can be detected by restriction analysis of the PCR product. As an additional control, substrate specificity of A3B and A3G was tested using lysates from 293T cells transiently transfected with A3B or A3G expression plasmids (**Supplementary Figure 5**). Of note, whereas the substrate TTCA (YTCA) was reported as a statistically favorable target for A3A over A3B in cancer tissues (Chan et al., 2015), TTC was demonstrated as a preferred target for all A3s except for A3G, *in vitro*, based on high resolution structures, protein–DNA interaction studies, and enzymatic assays (Yu et al., 2004; Harjes et al., 2017; Jaguva Vasudevan et al., 2017; Kouno et al., 2017; Shi et al., 2017; Yang et al., 2017). The assay demonstrated that A3G deaminase activity is present in all three UCCs (**Figure 4C**, CCCA panel). A3G deaminase activity was robust in 5637 cells, but lower in UMUC3 cells and barely detectable in VM-CUB1 cells. Importantly, as expected from the CCCA substrate specificity of A3G (Yu et al., 2004; Jaguva Vasudevan et al., 2017), siRNA-mediated knockdown of A3G affected product formation in the CCCA assay more efficiently than in the TTCA assay (**Figure 4C**). Using the CCCA substrate, A3B downregulation slightly reduced product formation, whereas simultaneous knockdown of A3B and A3G abolished detectable deaminase activity. Conversely, using the TTCA substrate, A3B knockdown, but not A3G knockdown resulted in complete loss of detectable deaminase activity (**Figure 4C**, TTCA panel). Taken together, these data confirm that A3G favors the CCCA sequence motif and A3B prefers the TTCA motif, but also indicate that A3B might mutate CCCA sequences on ssDNA substrates with a low frequency. More importantly, combining the deamination assay data (**Figure 4C**) with the A3 expression data presented in **Figures 1** and **4A** leads to the conclusion that *in vitro* A3B is the predominantly active member of the A3 family in the tested UCCs, whereas A3G-specific deaminase activity is comparably low.

Next, we investigated the effect of the expression of functional L1 elements on the deamination activity of A3 proteins by ectopically overexpressing transfected functional L1 elements encoded by pAJG101/L1_RP_ in UCCs. Lysates from transfected and untransfected UCCs were then either treated with RNase A to eliminate potential inhibitory RNA molecules, or left untreated, before they were assayed for A3B- or A3G-specific deaminase activity (Soros et al., 2007; McDougall and Smith, 2011). Ectopic L1 expression did not affect A3B- or A3G-encoded deaminase activity in any of the transfected UCC lines (**Figure 4D**).

### L1 Downregulation Reduces Cell Viability Irrespective of Apoptosis and Induces Senescence in UCC

**FIGURE 5:**
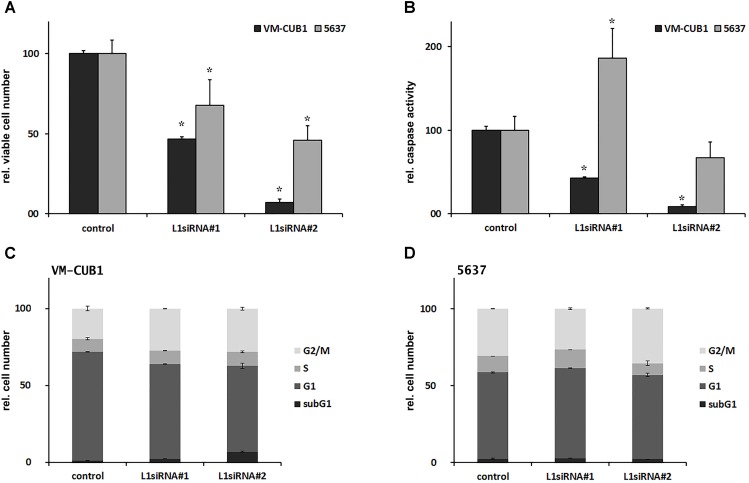
Phenotypical consequences of L1 siRNA treatment in selected UCCs. **(A)** Cell viability and **(B)** caspase activity (both depicted as percent of control) were determined in VM-CUB1 and 5637 UCCs after 72 h treatment with 20 nM control siRNA or L1 siRNAs (each *n* = 5–8). Cell cycle distribution in **(C)** VM-CUB1 and **(D)** 5637 cells (each *n* = 2). Data were represented as means ± standard deviations (error bars). *P*-values for **(A,B)** were calculated using Mann–Whitney *U* Test. Asterisk represents statistically significant difference: ^∗^*P* < 0.05 and ns, not significant.

While our results do not indicate that L1 expression affects A3B (or other A3) expression consistently, L1 silencing by siRNA impaired cell proliferation. In VM-CUB1 cells expressing L1 more strongly, the number of viable cells decreased to 47% and 7% after L1 knockdown using L1_siRNA#1 and L1_siRNA#2, respectively, compared to control siRNA-transfected cells (**Figure 5A**). The number of viable 5637 UCCs was less severely depleted to 68% and 46% after treatment with L1_siRNA#1 and L1_siRNA#2, respectively (**Figure 5A**). Caspase 3/7 activity, measured as an indicator of apoptosis, decreased to 43% and 8% in VM-CUB1 cells after L1_siRNA#1 and L1_siRNA#2 treatment, respectively (**Figure 5B**). In 5637 UCCs, caspase activity was increased after treatment with L1_siRNA#1, but not with L1_siRNA#2 (**Figure 5B**). According to flow cytometry data, the fraction of VM-CUB1 cells in G2/M phase was increased by roughly 8% in cells treated with either L1 siRNA (**Figure 5C**). In VM-CUB1 cells treated with L1_siRNA#2, the fraction of subG1 cells also decreased. S-phase fraction was unchanged (**Figure 5C**) and accordingly, incorporation of EdU was only slightly diminished, especially following L1_siRNA#1 treatment (**Supplementary Figure 6A**). Only minor changes in cell cycle distribution and, accordingly, EdU incorporation were seen in 5637 UCCs (**Figure 5D** and **Supplementary Figure 6A**). Thus, the decrease in viable cells following L1 knockdown does not reflect apoptosis.

In keeping with its effects on cell viability in short-term experiments, FL-L1 knockdown strongly diminished the clonogenicity of VM-CUB1 cells (**Supplementary Figure 6B**). Moreover, some VM-CUB1 cells showed morphological changes typical of senescent cells and stained positive for senescence-associated (SA)-β-galactosidase after treatment with either L1 siRNA, but very rarely after treatment with control siRNA (**Supplementary Figure 6D**). Unexpectedly, in 5637 cells, clonogenicity reproducibly increased after L1 knockdown using L1_siRNA#2, whereas L1_siRNA#1 exerted no significant effects on clonogenicity (**Supplementary Figure 6C**). Accordingly, no indications of senescence were detected in 5637 cells after treatment with L1 siRNAs or control siRNA (data not shown). Thus, L1 knockdown affected VM-CUB1 UCCs with high L1 expression levels more severely than 5637 cells with low L1 expression.

## Discussion

### The APOBEC3 Signature in Urothelial Carcinoma

Mutations induced by misdirected activity of A3 proteins have been implicated in several cancer types (Alexandrov et al., 2013; Burns et al., 2013; Lawrence et al., 2013; Roberts et al., 2013). Following viral replication or in the context of other genomic disturbances, A3 proteins can act as endogenous sources of mutations that can promote genomic instability in cancer evolution (Tubbs and Nussenzweig, 2017). The contribution of A3s is especially plausible in cancers elicited by viruses, such as cervical cancer (Henderson et al., 2014), but the high frequency of an APOBEC3-related mutational signature in UC (Alexandrov et al., 2013; Lawrence et al., 2013; Roberts et al., 2013) remains unexplained. Indeed, in a recently published molecular characterization of muscle-invasive bladder cancer, it was calculated that APOBEC3-mediated mutagenesis contributes 67% of all single nucleotide variants (SNVs) (Robertson et al., 2017).

### APOBEC Isoenzymes in Urothelial Carcinogenesis

A specific question is, which member of the A3 protein family is responsible for the observed mutational signature in UC. Bioinformatic analyses suggest that the mutational signature in UC matches better A3A than A3B specificity (Chan et al., 2015; Lamy et al., 2016). However, the expression of *A3B* was reported to exceed *A3A* expression in UC tissues (Lamy et al., 2016) and A3B may be more capable of introducing base substitutions in genomic DNA in human cells (Shinohara et al., 2012). Likewise, our results demonstrated robust upregulation of *A3B* expression in 16/17 UCC lines relative to normal urothelial cell cultures (**Figure 1**), whereas *A3A* was essentially undetectable in almost all (16/17) analyzed UCCs. Obviously, expression and enzymatic activity of A3 family members may vary during urothelial carcinogenesis and may not be fully reflected in the pattern seen in UCCs. However, A3 mutational activity was shown to be involved in both early and late mutation events that occurred during urothelial carcinogenesis arguing rather for continuous A3 mutational activity (McGranahan et al., 2015; Hurst et al., 2017; Robertson et al., 2017). Accordingly, *A3B* expression levels exceeding *A3A* were also observed in high-grade non-muscle invasive bladder cancers (NMIBCs) (Hedegaard et al., 2016). Furthermore, it seems unlikely that A3A would be selectively repressed in UCCs, whereas A3B remains upregulated. Thus, our results rather argue for the enzymatic activity of A3B being responsible for the observed mutations, at least in the context of UCC lines. Conceivably, A3A expression in UC tissues may partly result from macrophages and monocytes highly prevalent in high-grade NMIBCs (Peng et al., 2007; Koning et al., 2009; Thielen et al., 2010; Takeuchi et al., 2016), or may be induced in UC cells *in vivo* by factors located in the tumor environment. Currently available antibodies directed against A3B cannot detect A3B at levels present in UCC lines (Burns et al., 2015; Jaguva Vasudevan et al., 2018). However, since we could demonstrate that the amounts of expressed A3G proteins correspond to their *A3G* mRNA levels (**Figures 1**, **4B**) in UCCs 5637, UMUC3 and VM-CUB1, this is very likely to be the case for A3B too. Moreover, cytidine deamination assays coupled with knockdown experiments convincingly revealed the expected substrate-specific activity levels for both A3B and A3G. Of note, the general DNA motif reported to be recognized by APOBEC proteins to introduce somatic mutations in cancer is “TC” (Roberts et al., 2013) (the A3B-specific motif in our assay here is TTCA). However, A3G recognizes the DNA sequence motif (CCCA) (Jaguva Vasudevan et al., 2013; Yang et al., 2017). In addition, A3G reportedly possesses a cytoplasmic retention signal that retains A3G exclusively in the cytoplasm (Jaguva Vasudevan et al., 2013; Bennett et al., 2008). For these reasons, A3G is not considered to contribute to A3-mediated mutagenesis during carcinogenesis. Interestingly, A3G may influence cancer cell survival via its likely role in DSB repair (Nowarski and Kotler, 2013).

### Are There Any Effects of Endogenous L1 Activity on A3 Upregulation in Urothelial Cancer Cells?

To address the general question of what triggers A3 activation in urothelial cancer cells, we pursued the hypothesis that A3 activation may be elicited by endogenous retroelement activity rather than the presence of exogenous viruses. Expression of functional endogenous L1 elements seems a plausible cause for A3 activation, because in urothelial cancer cells, L1 promoter sequences are frequently hypomethylated, and FL-L1 expression is increased even more than in other cancer types (Kreimer et al., 2013; Nusgen et al., 2015). In comparison, neither *Alu* nor HERV-K sequences are significantly upregulated in UCCs (Kreimer et al., 2013). However, our combined results do not allow drawing the conclusion that L1 activity is a major factor for A3 activation as neither siRNA-mediated downregulation of endogenous FL-L1 elements nor ectopic overexpression of RC-L1 reporter elements led to any consistent and significant alteration in the expression of any A3 protein family member. Only in VM-CUB1 cells the overexpression of the L1 reporter plasmid pAJG101/L1_RP_ led to a significant increase of A3B transcript levels (**Figure 3**). In addition, endogenous FL-L1 and *A3* expression levels did not correlate with each other across the tested panel of cell lines. Here, future investigations are required to unambiguously elucidate any role of L1 expression and/or retrotransposition activity in the activation of A3 proteins in tumor cells.

For instance, it might be useful to investigate the effects of the codon-optimized L1 element, *ORFeus-Hs* (An et al., 2011) that produces 5- to 10-fold more L1 proteins than the L1_RP_ element used in our study, on the expression of endogenous APOBEC3 gene products.

Knocking down the expression of endogenous FL-L1 elements with two different siRNAs targeting the intact ORF1 coding region resulted in the efficient depletion of endogenous L1 ORF1p. This observation indicates that the majority of transcripts from active L1Hs elements harboring intact ORF1 sequences were removed from the tested cell lines. However, these siRNAs did not decrease the overall FL-L1 transcript levels as measured by RT-qPCR to the same degree (**Figure 2**). This could be explained by the fact that the L1 5′UTR-specific primers used for the RT-qPCR assay also detect transcripts from FL-L1 elements with non-functional ORF1 sequences, which are not or less efficiently targeted by the siRNAs.

In future work, it should be worthwhile investigating the impact of siRNAs targeting also non-functional L1 transcripts on A3 expression as well.

Although we did not observe any effect of L1 repression on A3 activity, it is obviously capable to elicit severe effects in UCCs. In particular, efficient knockdown of ORF1p expressing FL-L1 elements by siRNAs diminished proliferation of UCCs with higher L1 expression levels (such as VM-CUB1), but had less effect on UCCs exhibiting lower L1 expression levels (such as 5637 cells). These results are in good agreement with previous reports that L1 knockdown causes a loss of proliferative ability in tumor cells independent from apoptosis (Aschacher et al., 2016), ultimately leading to senescence (Oricchio et al., 2007; Sciamanna et al., 2014; Aschacher et al., 2016). However, this issue has not been investigated in UCCs previously. Since L1 activation may be particularly prevalent in UC (Nusgen et al., 2015; Whongsiri et al., 2018), this result calls for closer investigations of L1 function in UC carcinogenesis, beyond retrotransposition. There is growing evidence suggesting that expression and retrotransposition of LINE-1 in neoplasms affects transcription initiation of oncogenes (Rodic and Burns, 2013). Also in hepatocellular carcinoma, L1 ORF1p was suggested to promote cell proliferation and resistance to chemotherapy (Feng et al., 2013). Indeed, L1 expression is linked to the activation of epithelial-mesenchymal transition (EMT) and was shown to affect the expression of miRNA genes (let-7 miRNA family) specifically regulating tumor suppressor expression (Rangasamy et al., 2015). Consistently, our study found cell growth impairment as a consequence of L1 silencing in UCCs, which requires further studies to identify any specific factor(s) or pathway that is involved in this regulation.

### Potential Causes of APOBEC Activation in Urothelial Carcinoma

Finally, if there is no evidence that A3 activation in UC is elicited by either exogenous virus infection or endogenous L1 retrotransposon activation, what causes it? Several alternative hypotheses deserve investigation. For instance, A3B is induced by several cytokines in normal liver (Lucifora et al., 2014) and through the PKC-NFκB signaling pathway in several cancers (Leonard et al., 2015). These factors may also be relevant in urothelial carcinogenesis and could be fostered by a persistent inflammatory state (Thompson et al., 2015). Interestingly, a recent analysis of A3 expression in UC tissues by Glaser et al. (2018) revealed rather uniform expression of A3B in various molecular subtypes of the disease, whereas A3A was mostly expressed in the basal, squamous-like subtype. A3-high tumors demonstrated higher expression of relevant immune marker genes. A3 genes are inducible by interferon and thus belong to the group of interferon-stimulated genes (ISGs). Indeed, Glaser et al. (2018) could induce A3B expression in the UC cell lines HT-1376 and UMUC3 by IFNγ treatment, but not in two cell lines with initially low A3B expression. Unfortunately, they did not report on A3A expression in UC cell lines.

Most advanced muscle-invasive UCs contain mutations inactivating p53, which are rare in non-muscle invasive UC (Hurst et al., 2017; Robertson et al., 2017). The p53 tumor suppressor also regulates the transcription of several A3 genes. In particular, loss of p53 or overexpression of gain-of-function mutants leads to upregulation of A3B (Menendez et al., 2017; Periyasamy et al., 2017). Loss of p53 function may therefore contribute to A3B activation in muscle-invasive UC, but not likely in non-muscle invasive tumors.

Moreover, recent results suggest that A3B may target ssDNA accumulating as a result of replication stress (Kanu et al., 2016) or transcription stress (Periyasamy et al., 2015; Tubbs and Nussenzweig, 2017). ssDNA formed preferentially during lagging strand synthesis in the course of DNA replication and displaced non-transcribed strand ssDNA due to transcription overload, e.g., as a result of hormone stimulation (Periyasamy et al., 2015; Haradhvala et al., 2016; Hoopes et al., 2016). Indeed, replication stress is thought to be common during urothelial carcinogenesis (Schepeler et al., 2013) and exacerbated by p53 loss of function.

Thus, we conclude that several factors may cooperate to activate A3 in urothelial carcinogenesis. This work largely excludes the pervasive activation of L1 retroelements as one potential factor. Moreover, in line with some, but not other previous reports, detailed analysis of UCCs suggests A3B rather than A3A as the predominantly active enzyme.

The major limitations of our study concern the detection of A3 proteins and the high L1 copy number in the human genome. With respect to A3 proteins, we could not obtain antibodies that are sufficiently sensitive and specific to detect endogenous expression of each isoenzyme. A reliable array of such antibodies would be very helpful to characterize the expression pattern of A3s in UC cell lines and tissues more precisely. Also the highly repetitive character of endogenous L1 retroelements causes major complications for our studies. To fully understand the impact of L1 activity in UC cells and tissues, a complete characterization of the repertoire of transcripts from retrotransposition-competent L1 elements and, ideally, from non-functional L1 elements will be required. Third generation techniques currently under development will hopefully enable this investigation.

## Author Contributions

WG, WS, AAJV, and CM conceived and designed the experiments. AV and WG performed most of the experiments. GS performed immunoblot analyses, generated the L1 reporter plasmid pAJG101/L1_RP,_ and participated in drafting the manuscript. UK and AK performed some experiments. WG, WS, AAJV, DH, and CM analyzed the data. WS, GS, and CM contributed reagents and tools. WG, WS, AAJV, GS, and CM wrote the paper.

## Conflict of Interest Statement

The authors declare that the research was conducted in the absence of any commercial or financial relationships that could be construed as a potential conflict of interest.
