# Supplementary material for: APOBEC3B Activity Is Prevalent in Urothelial Carcinoma Cells and Only Slightly Affected by LINE-1 Expression
Source: Front Microbiol. 2018 Sep 4;9:2088. doi: 10.3389/fmicb.2018.02088 (PMC6132077; doi:10.3389/fmicb.2018.02088)
Supplement: Supplementary file 1 [file Table_1.DOCX]

**Suppl. Table 1:** **Removal of genomic DNA from RNA preparations of selected UCCs.** To evaluate the efficiency of DNA wipeout reaction provided with the QuantiTect Reverse Transcription Kit (Qiagen, Hilden, Germany), 1-µl aliquot of the DNAse-treated RNA sample was taken prior to cDNA synthesis to use it for PCR without cDNA synthesis (no-RT sample). The remaining RNA was subjected to RT-mediated cDNA synthesis. qPCR analysis of no-RT sample as well as cDNA sample was performed with equivalent amounts of input nucleic acids (equivalent RNA and cDNA concentrations were used, *see methods*). Water served as blank. “NOAMP” represents failed amplification within the 40 cycles.

| **Cell lines** | **RNA after genomic DNA removal (no RT)**  **Input: 4.64 ng/μl RNA**  **[Ct value]** | | **cDNA from respective RNA after genomic DNA removal (+RT)**  **Input: 4.64 ng/μl cDNA**  **[Ct value]** | |
| --- | --- | --- | --- | --- |
| **5637** | NOAMP | NOAMP | 21.23 | 21.22 |
| **VM-CUB1** | 33.50 | NOAMP | 20.14 | 20.13 |
| **639V** | 38.21 | 35.00 | 20.12 | 20.29 |
| **SD** | 35.76 | 34.39 | 20.52 | 20.51 |
| **BC61** | 35.54 | NOAMP | 20.20 | 20.21 |
| **RT4** | 35.66 | 35.27 | 21.16 | 21.12 |
| **Blank (water)** | 35.30 and 34.27 | | | |
