# Supplementary material for: APOBEC3B Activity Is Prevalent in Urothelial Carcinoma Cells and Only Slightly Affected by LINE-1 Expression
Source: Front Microbiol. 2018 Sep 4;9:2088. doi: 10.3389/fmicb.2018.02088 (PMC6132077; doi:10.3389/fmicb.2018.02088)
Supplement: Supplementary file 4 [file Presentation_1.pptx]

## Slide 1
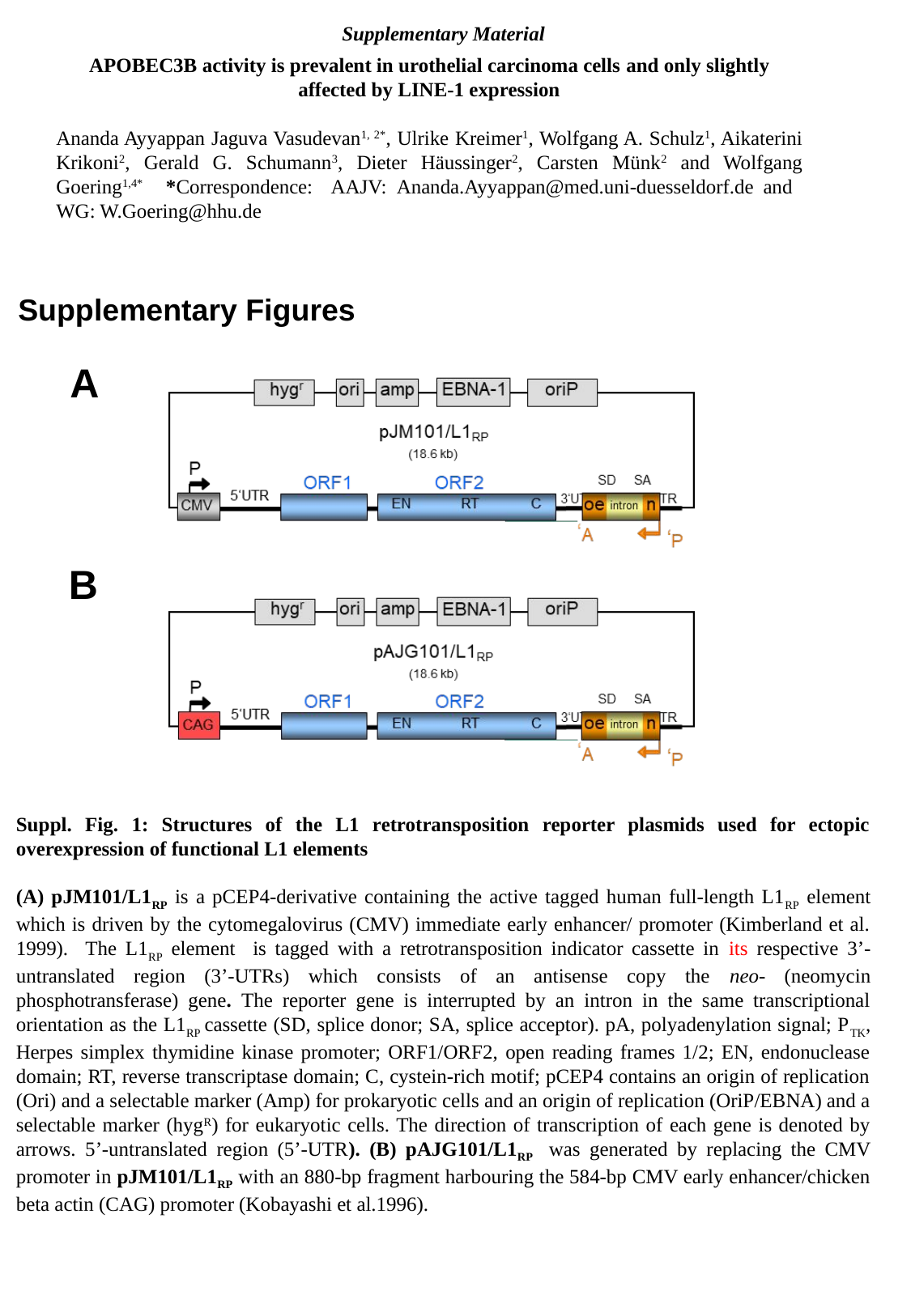

Supplementary Material
APOBEC3B activity is prevalent in urothelial carcinoma cells and only slightly affected by LINE-1 expression
Ananda Ayyappan Jaguva Vasudevan1, 2*, Ulrike Kreimer1, Wolfgang A. Schulz1, Aikaterini Krikoni2, Gerald G. Schumann3, Dieter Häussinger2, Carsten Münk2 and Wolfgang Goering1,4* *Correspondence: AAJV: Ananda.Ayyappan@med.uni-duesseldorf.de and WG: W.Goering@hhu.de
Supplementary Figures
A
B
Suppl. Fig. 1: Structures of the L1 retrotransposition reporter plasmids used for ectopic overexpression of functional L1 elements
(A) pJM101/L1RP is a pCEP4-derivative containing the active tagged human full-length L1RP element which is driven by the cytomegalovirus (CMV) immediate early enhancer/ promoter (Kimberland et al. 1999). The L1RP element is tagged with a retrotransposition indicator cassette in its respective 3’-untranslated region (3’-UTRs) which consists of an antisense copy the neo- (neomycin phosphotransferase) gene. The reporter gene is interrupted by an intron in the same transcriptional orientation as the L1RP cassette (SD, splice donor; SA, splice acceptor). pA, polyadenylation signal; PTK, Herpes simplex thymidine kinase promoter; ORF1/ORF2, open reading frames 1/2; EN, endonuclease domain; RT, reverse transcriptase domain; C, cystein-rich motif; pCEP4 contains an origin of replication (Ori) and a selectable marker (Amp) for prokaryotic cells and an origin of replication (OriP/EBNA) and a selectable marker (hygR) for eukaryotic cells. The direction of transcription of each gene is denoted by arrows. 5’-untranslated region (5’-UTR). (B) pAJG101/L1RP was generated by replacing the CMV promoter in pJM101/L1RP with an 880-bp fragment harbouring the 584-bp CMV early enhancer/chicken beta actin (CAG) promoter (Kobayashi et al.1996).

## Slide 2
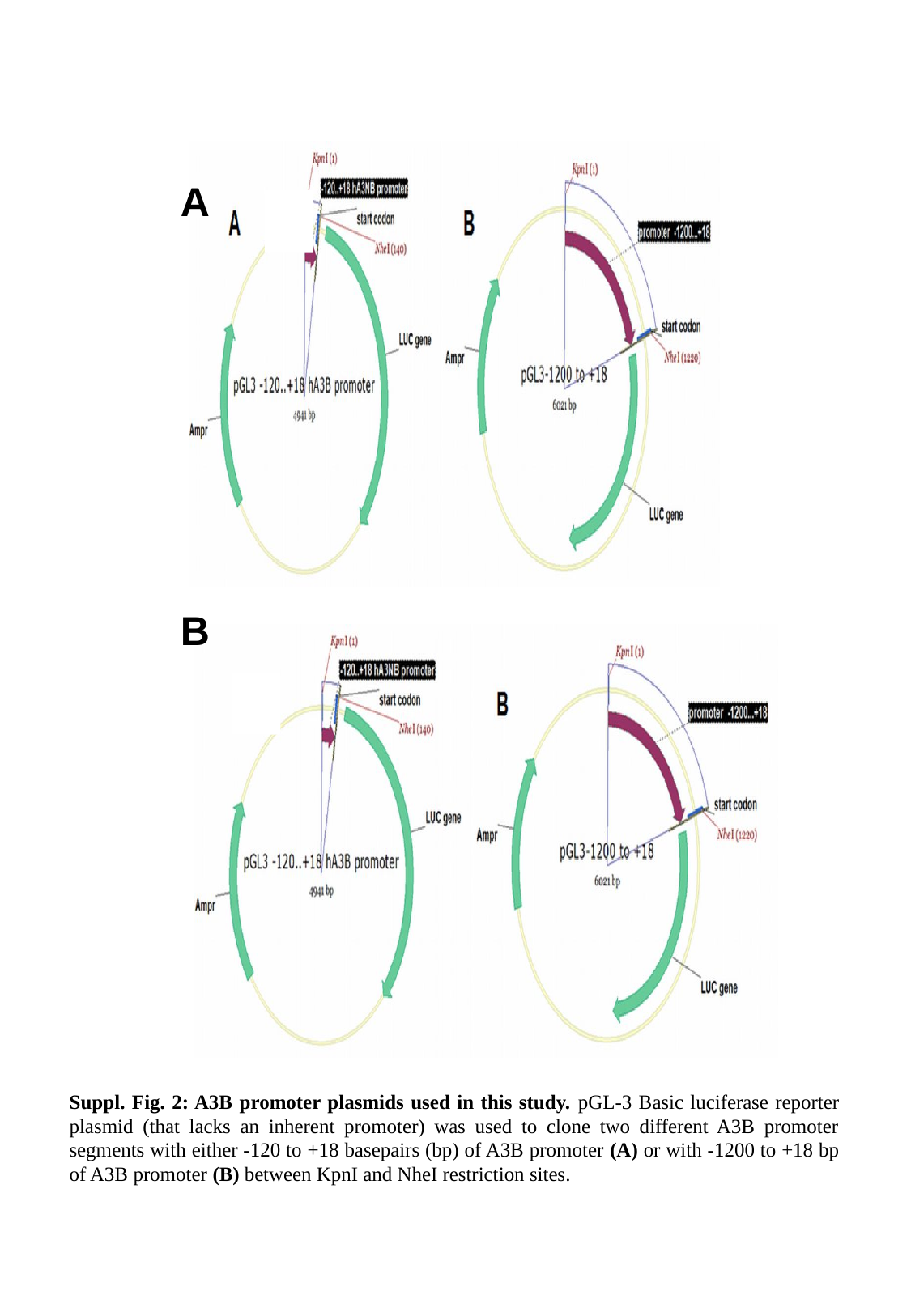

A
B
Suppl. Fig. 2: A3B promoter plasmids used in this study. pGL-3 Basic luciferase reporter plasmid (that lacks an inherent promoter) was used to clone two different A3B promoter segments with either -120 to +18 basepairs (bp) of A3B promoter (A) or with -1200 to +18 bp of A3B promoter (B) between KpnI and NheI restriction sites.

## Slide 3
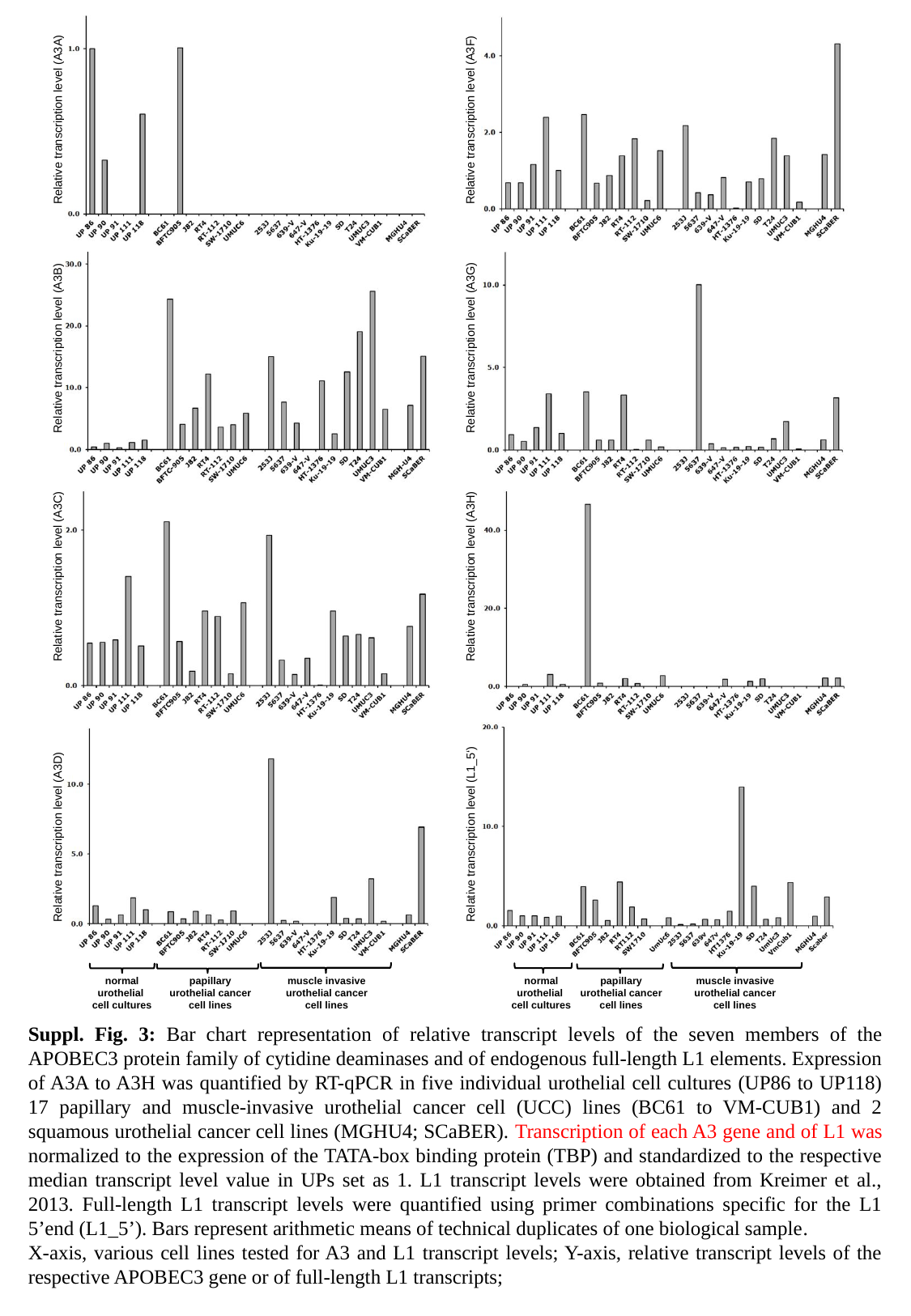

Relative transcription level (A3F)
Relative transcription level (A3A)
Relative transcription level (A3B)
Relative transcription level (A3G)
Relative transcription level (A3C)
Relative transcription level (A3H)
Relative transcription level (A3D)
Relative transcription level (L1_5‘)
normal urothelial
cell cultures
papillary urothelial cancer cell lines
muscle invasive urothelial cancer cell lines
normal urothelial
cell cultures
papillary urothelial cancer cell lines
muscle invasive urothelial cancer cell lines
Suppl. Fig. 3: Bar chart representation of relative transcript levels of the seven members of the APOBEC3 protein family of cytidine deaminases and of endogenous full-length L1 elements. Expression of A3A to A3H was quantified by RT-qPCR in five individual urothelial cell cultures (UP86 to UP118) 17 papillary and muscle-invasive urothelial cancer cell (UCC) lines (BC61 to VM-CUB1) and 2 squamous urothelial cancer cell lines (MGHU4; SCaBER). Transcription of each A3 gene and of L1 was normalized to the expression of the TATA-box binding protein (TBP) and standardized to the respective median transcript level value in UPs set as 1. L1 transcript levels were obtained from Kreimer et al., 2013. Full-length L1 transcript levels were quantified using primer combinations specific for the L1 5’end (L1_5’). Bars represent arithmetic means of technical duplicates of one biological sample.
X-axis, various cell lines tested for A3 and L1 transcript levels; Y-axis, relative transcript levels of the respective APOBEC3 gene or of full-length L1 transcripts;

## Slide 4
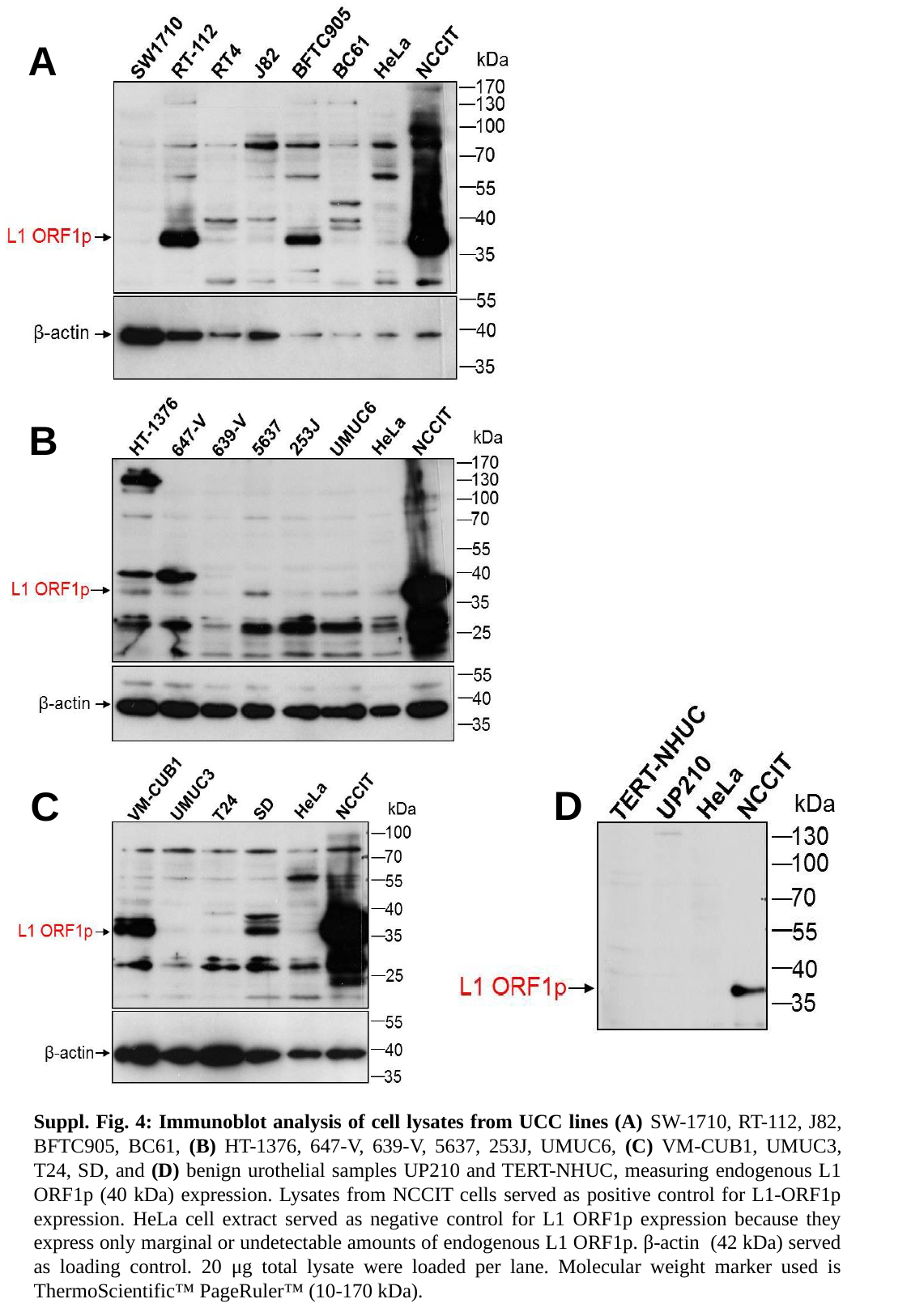

A
D
C
B
Suppl. Fig. 4: Immunoblot analysis of cell lysates from UCC lines (A) SW-1710, RT-112, J82, BFTC905, BC61, (B) HT-1376, 647-V, 639-V, 5637, 253J, UMUC6, (C) VM-CUB1, UMUC3, T24, SD, and (D) benign urothelial samples UP210 and TERT-NHUC, measuring endogenous L1 ORF1p (40 kDa) expression. Lysates from NCCIT cells served as positive control for L1-ORF1p expression. HeLa cell extract served as negative control for L1 ORF1p expression because they express only marginal or undetectable amounts of endogenous L1 ORF1p. β-actin (42 kDa) served as loading control. 20 μg total lysate were loaded per lane. Molecular weight marker used is ThermoScientific™ PageRuler™ (10-170 kDa).

## Slide 5
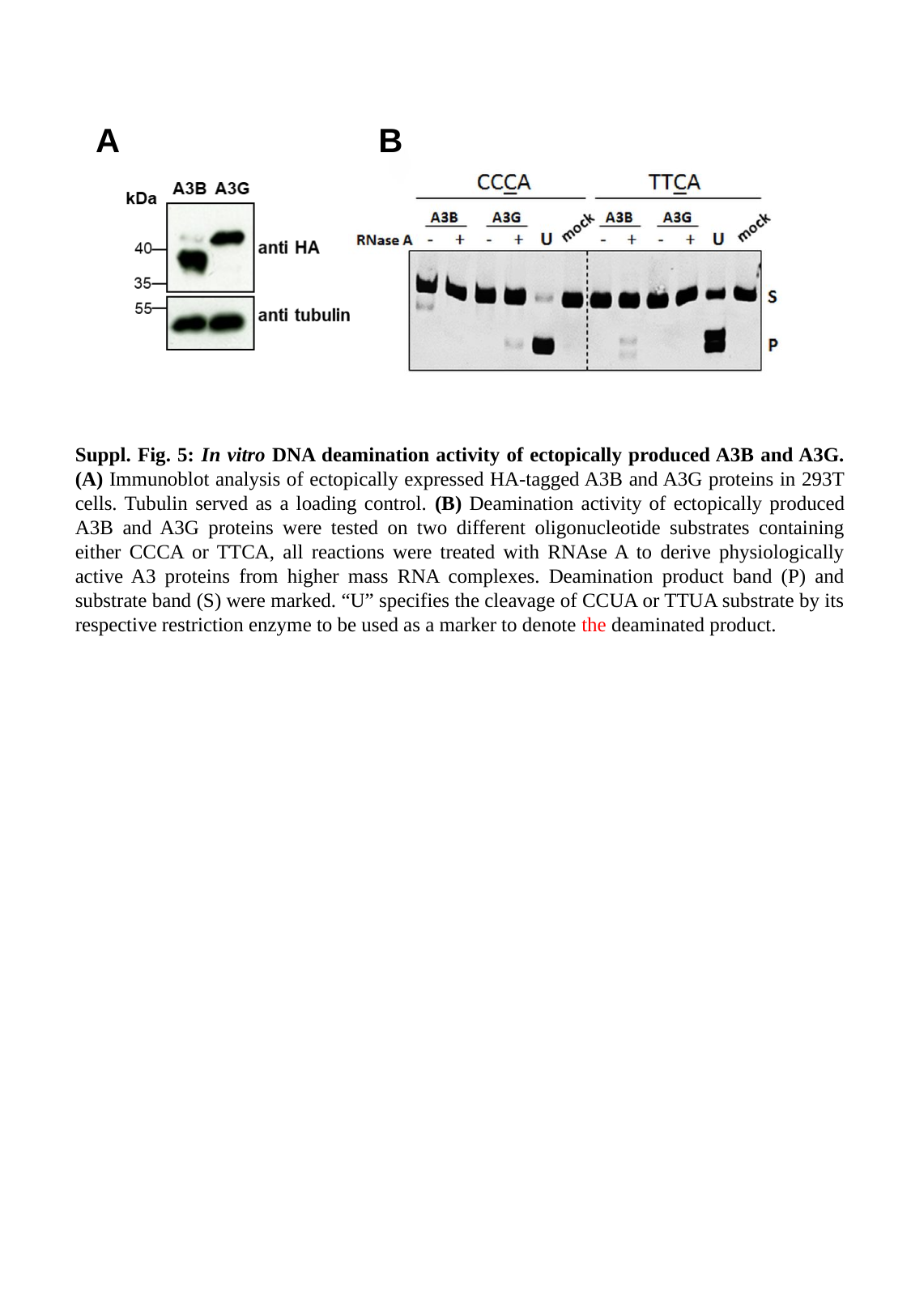

A
B
Suppl. Fig. 5: In vitro DNA deamination activity of ectopically produced A3B and A3G. (A) Immunoblot analysis of ectopically expressed HA-tagged A3B and A3G proteins in 293T cells. Tubulin served as a loading control. (B) Deamination activity of ectopically produced A3B and A3G proteins were tested on two different oligonucleotide substrates containing either CCCA or TTCA, all reactions were treated with RNAse A to derive physiologically active A3 proteins from higher mass RNA complexes. Deamination product band (P) and substrate band (S) were marked. “U” specifies the cleavage of CCUA or TTUA substrate by its respective restriction enzyme to be used as a marker to denote the deaminated product.

## Slide 6
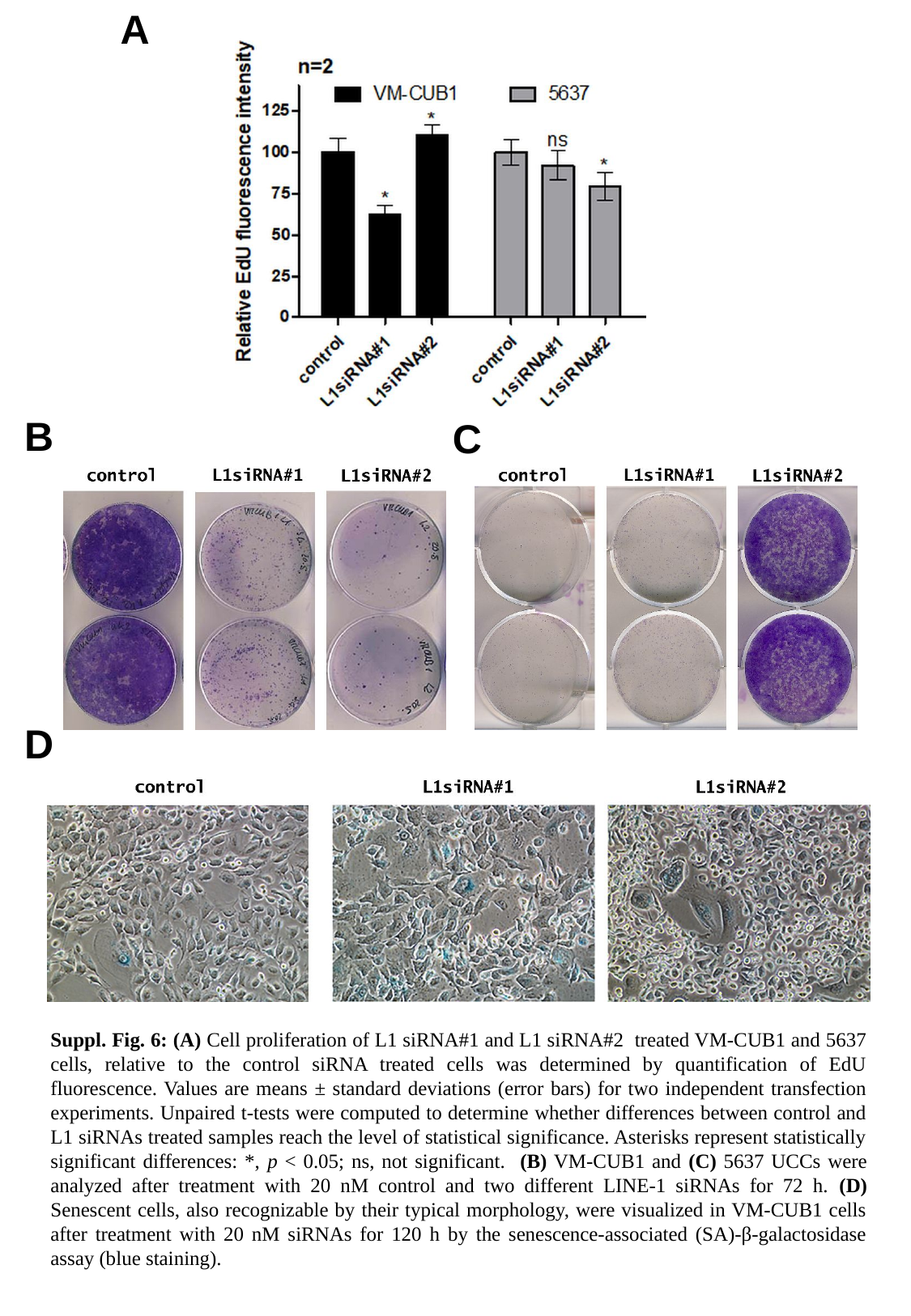

A
B
C
D
Suppl. Fig. 6: (A) Cell proliferation of L1 siRNA#1 and L1 siRNA#2 treated VM-CUB1 and 5637 cells, relative to the control siRNA treated cells was determined by quantification of EdU fluorescence. Values are means ± standard deviations (error bars) for two independent transfection experiments. Unpaired t-tests were computed to determine whether differences between control and L1 siRNAs treated samples reach the level of statistical significance. Asterisks represent statistically significant differences: *, p < 0.05; ns, not significant. (B) VM-CUB1 and (C) 5637 UCCs were analyzed after treatment with 20 nM control and two different LINE-1 siRNAs for 72 h. (D) Senescent cells, also recognizable by their typical morphology, were visualized in VM-CUB1 cells after treatment with 20 nM siRNAs for 120 h by the senescence-associated (SA)-β-galactosidase assay (blue staining).

## Slide 7
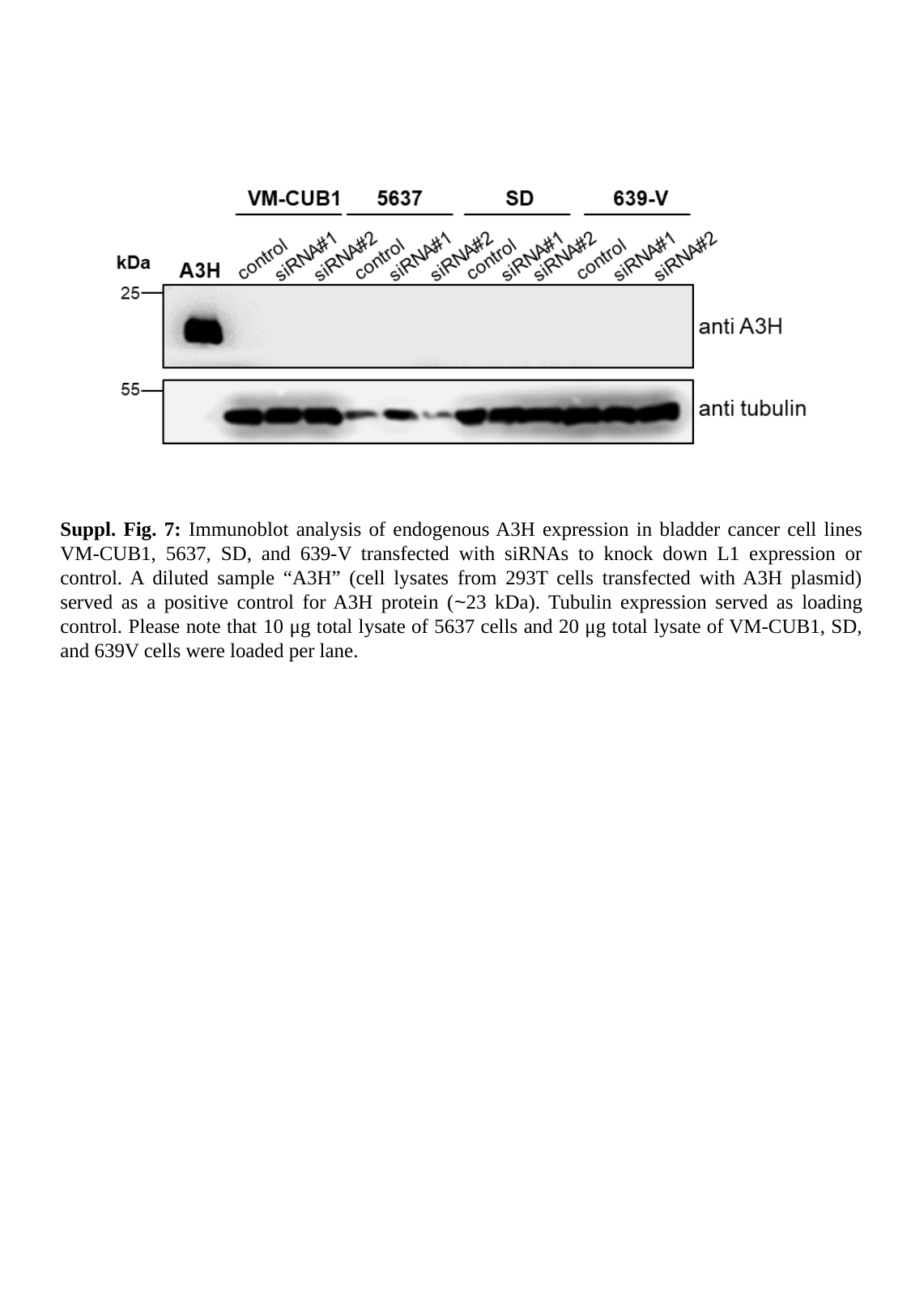

Suppl. Fig. 7: Immunoblot analysis of endogenous A3H expression in bladder cancer cell lines VM-CUB1, 5637, SD, and 639-V transfected with siRNAs to knock down L1 expression or control. A diluted sample “A3H” (cell lysates from 293T cells transfected with A3H plasmid) served as a positive control for A3H protein (~23 kDa). Tubulin expression served as loading control. Please note that 10 μg total lysate of 5637 cells and 20 μg total lysate of VM-CUB1, SD, and 639V cells were loaded per lane.

## Slide 8
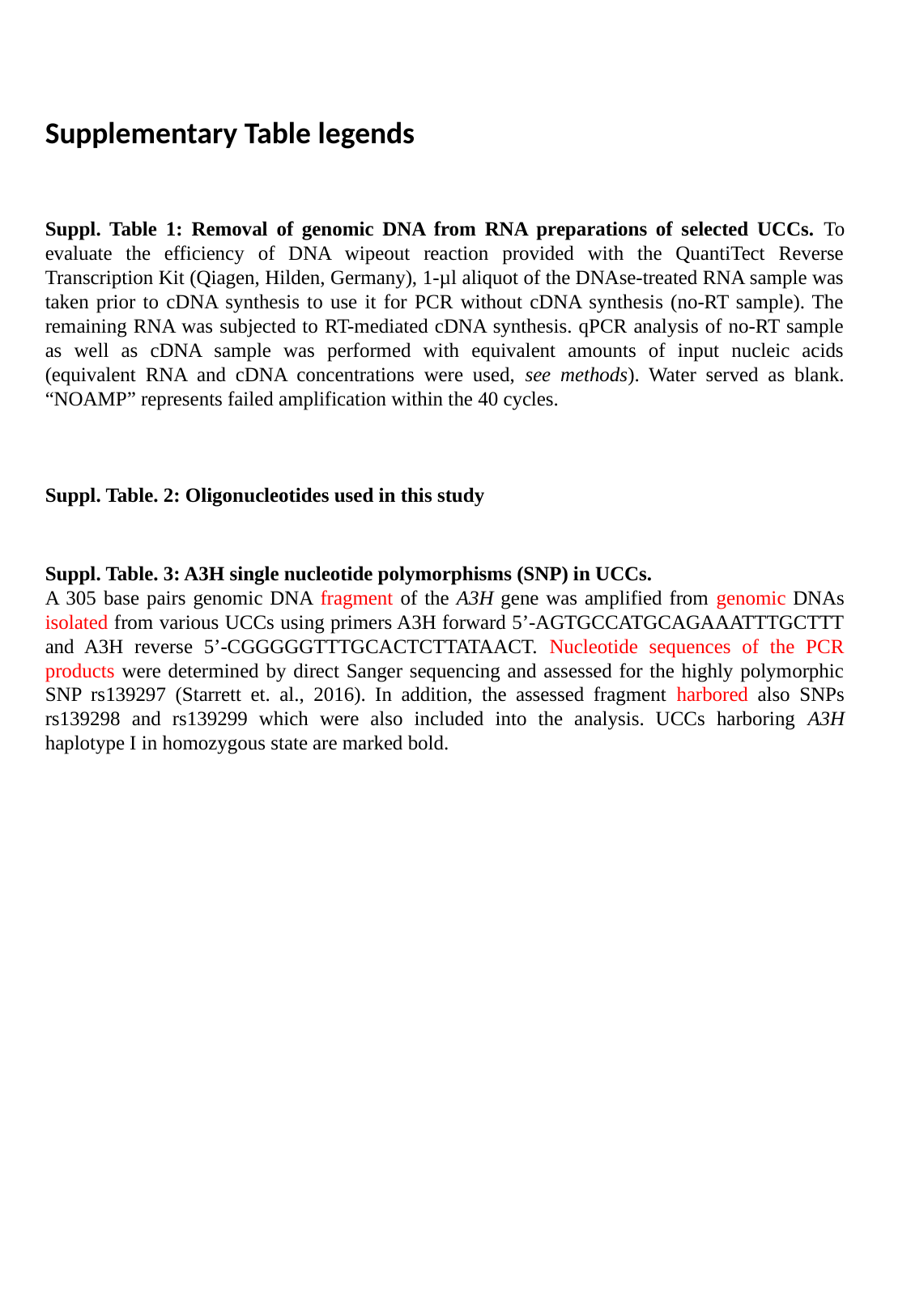

Supplementary Table legends
Suppl. Table 1: Removal of genomic DNA from RNA preparations of selected UCCs. To evaluate the efficiency of DNA wipeout reaction provided with the QuantiTect Reverse Transcription Kit (Qiagen, Hilden, Germany), 1-µl aliquot of the DNAse-treated RNA sample was taken prior to cDNA synthesis to use it for PCR without cDNA synthesis (no-RT sample). The remaining RNA was subjected to RT-mediated cDNA synthesis. qPCR analysis of no-RT sample as well as cDNA sample was performed with equivalent amounts of input nucleic acids (equivalent RNA and cDNA concentrations were used, see methods). Water served as blank. “NOAMP” represents failed amplification within the 40 cycles.
Suppl. Table. 2: Oligonucleotides used in this study
Suppl. Table. 3: A3H single nucleotide polymorphisms (SNP) in UCCs.
A 305 base pairs genomic DNA fragment of the A3H gene was amplified from genomic DNAs isolated from various UCCs using primers A3H forward 5’-AGTGCCATGCAGAAATTTGCTTT and A3H reverse 5’-CGGGGGTTTGCACTCTTATAACT. Nucleotide sequences of the PCR products were determined by direct Sanger sequencing and assessed for the highly polymorphic SNP rs139297 (Starrett et. al., 2016). In addition, the assessed fragment harbored also SNPs rs139298 and rs139299 which were also included into the analysis. UCCs harboring A3H haplotype I in homozygous state are marked bold.

## Slide 9
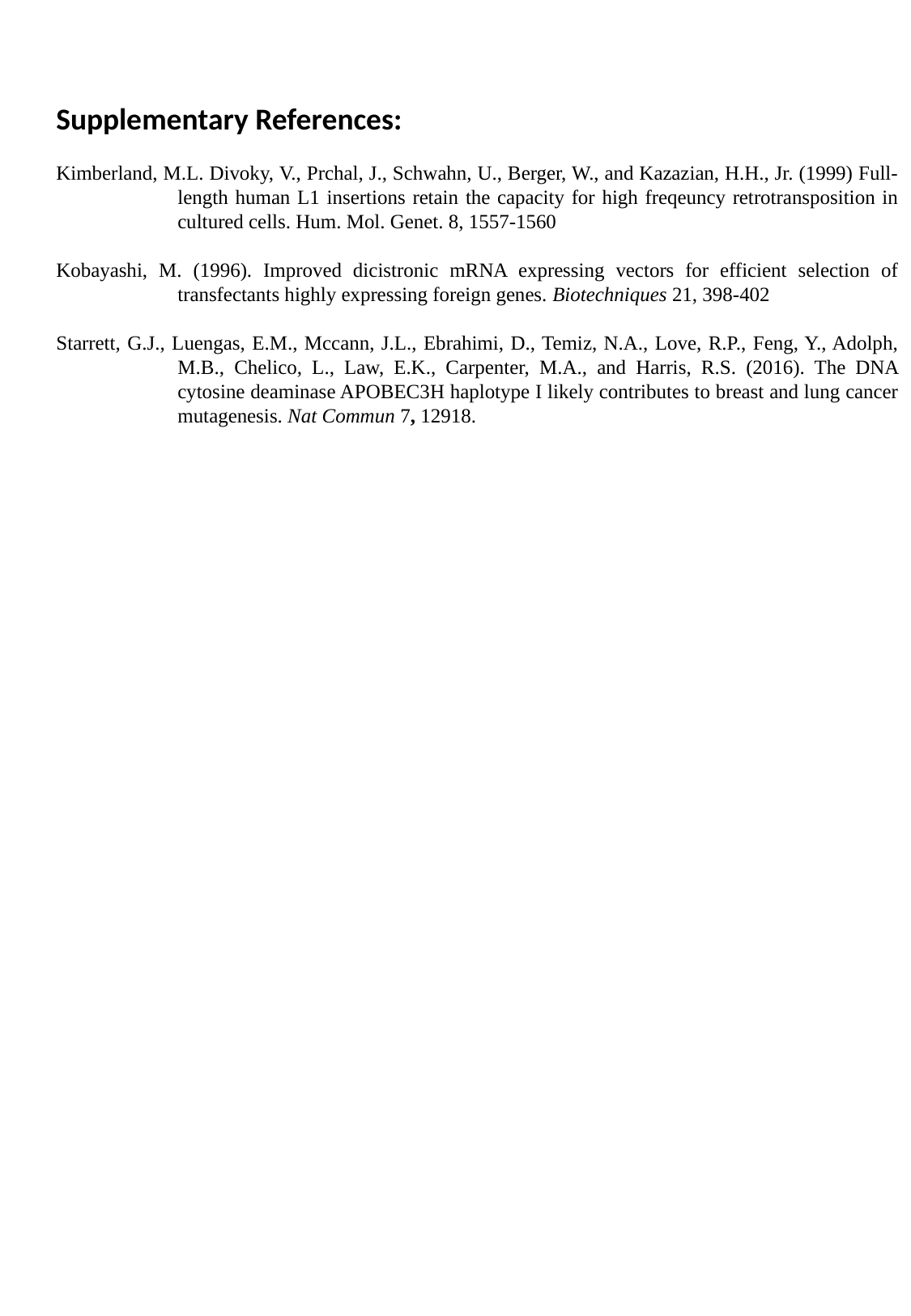

Supplementary References:
Kimberland, M.L. Divoky, V., Prchal, J., Schwahn, U., Berger, W., and Kazazian, H.H., Jr. (1999) Full-	length human L1 insertions retain the capacity for high freqeuncy retrotransposition in 	cultured cells. Hum. Mol. Genet. 8, 1557-1560
Kobayashi, M. (1996). Improved dicistronic mRNA expressing vectors for efficient selection of 	transfectants highly expressing foreign genes. Biotechniques 21, 398-402
Starrett, G.J., Luengas, E.M., Mccann, J.L., Ebrahimi, D., Temiz, N.A., Love, R.P., Feng, Y., Adolph, 	M.B., Chelico, L., Law, E.K., Carpenter, M.A., and Harris, R.S. (2016). The DNA 	cytosine deaminase APOBEC3H haplotype I likely contributes to breast and lung cancer 	mutagenesis. Nat Commun 7, 12918.
